# Process Accumulated 8% Efficient Cu_2_ZnSnS_4_‐BiVO_4_ Tandem Cell for Solar Hydrogen Evolution with the Dynamic Balance of Solar Energy Storage and Conversion

**DOI:** 10.1002/advs.202205726

**Published:** 2022-12-20

**Authors:** Hongwei Cai, Weidong Zhao, Guohong Xiao, Yucheng Hu, Xiaomin Wu, Huanyang Ni, Shigeru Ikeda, Yunhau Ng, Jiahua Tao, Lingzhi Zhao, Feng Jiang

**Affiliations:** ^1^ Institute of Hydrogen Energy for Carbon Peaking and Carbon Neutralization School of Semiconductor Science and Technology South China Normal University Foshan 528225 China; ^2^ Donghai Laboratory Zhoushan Zhejiang 316021 China; ^3^ Chengfeng Light Energy Science and Technology (Guangzhou) Limited Company Huangpu District Guangzhou 510670 China; ^4^ Key Laboratory of Polar Materials and Devices, Ministry of Education East China Normal University Information Building 500 Dongchuan Road Shanghai 200241 China; ^5^ Department of Chemistry Konan University 9‐1 Okamoto, Higashinada Kobe Hyogo 658‐8501 Japan; ^6^ School of Energy and Environment City University of Hong Kong Kowloon Hong Kong 999077 China

**Keywords:** greenhouse, light soaking effect, process accumulated, solar thermal energy storage and conversion, record solar to hydrogen efficiency, TD‐CZTS‐BiVO_4_ tandem cells

## Abstract

A process accumulated record solar to hydrogen (STH) conversion efficiency of 8% is achieved on the Cu_2_ZnSnS_4_‐BiVO_4_ tandem cell by the synergistic coupling effect of solar thermal and photoelectrochemical (PEC) water splitting with the dynamic balance of solar energy storage and conversion of the greenhouse system. This is the first report of a Cu_2_ZnSnS_4_‐BiVO_4_ tandem cell with a high unbiased STH efficiency of over 8% for solar water splitting due to the greenhouse device system. The greenhouse acts as a solar thermal energy storage cell, which absorbs infrared solar light and storage as thermal energy with the solar light illumination time, while thermoelectric device (TD) converts thermal energy into electric power, electric power is also recycled and added onto Cu_2_ZnSnS_4_‐BiVO_4_ tandem cell for enhanced overall water splitting. Finally, the solar water splitting properties of the TD‐Cu_2_ZnSnS_4_‐BiVO_4_ integrated tandem cell in pure natural seawater are demonstrated, and a champion STH efficiency of 2.46% is presented, while a large area (25 cm^2^) TD‐Cu_2_ZnSnS_4_‐BiVO_4_ integrated tandem device with superior long‐term stability is investigated for 1 week, which provides new insight into photoelectrochemical solar water splitting devices.

## Graphical abstract

A process accumulated record solar to hydrogen (STH) conversion efficiency of 8% is achieved on the Cu_2_ZnSnS_4_‐BiVO_4_ tandem cell by the synergistic coupling effect of solar thermal and photoelectrochemical water splitting with the dynamic balance of solar energy storage and conversion of the greenhouse system.

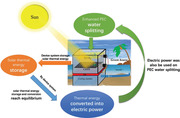

## 1 Introduction

Energy shortages and environmental pollution have become important issues that urgently need to be solved for social development, and people have to actively look for other sustainable energy sources to cope with survival challenges for the future.^[^ ^1^, ^2^, ^3^, ^4^, ^5^ ^]^ Inspired by natural photosynthesis, photoelectrochemical (PEC) solar water splitting is an attractive technology to convert solar energy into hydrogen, which is a storable and clean chemical fuel.^[^ ^6^, ^7^, ^8^, ^9^, ^10^, ^11^ ^]^ Some photosensitivity semiconductor materials, such as Si, BiVO_4_, TiO_2_, GaN, Cu(In,Ga)Se_2_, CdTe, GaAs, Sb_2_Se_3_, and so on, have been applied to PEC water splitting and showed remarkable hydrogen evolution performance.^[^ ^12^, ^13^, ^14^, ^15^, ^16^, ^17^, ^18^, ^19^, ^20^, ^21^, ^22^, ^23^ ^]^ Nevertheless, limited by the complex process and expensive raw materials of refining silicon, the rarity of indium in Cu(In,Ga)Se_2_, and the toxicity of cadmium in CdTe, researchers are looking for new low‐cost and environmentally friendly semiconductor materials to substitute for materials such as Si, Cu(In,Ga)Se_2_, and CdTe.^[^ ^24^, ^25^, ^26^, ^27^, ^28^, ^29^, ^30^, ^31^ ^]^ Fortunately, the low‐cost, environmentally friendly, and earth‐abundant kesterite Cu_2_ZnSnS_4_ (CZTS) with an appropriate band gap (1.45 eV) and excellent light harvesting properties meets our needs for PEC solar water splitting.^[^ ^32^, ^33^, ^34^, ^35^, ^36^, ^37^, ^38^, ^39^, ^40^ ^]^ In 2010, Yokoyama et al. successfully prepared a CZTS thin film and applied it to PEC solar water splitting. A CdS layer was deposited onto the CZTS to accelerate the separation of photogenerated carriers by forming a space electric field at the interface of the CdS/CZTS.^[^ ^41^ ^]^ Subsequently, Ma and Wang et al. used a Mo grid‐modified substrate as the back contact for CZTS‐based photocathodes and modified it with Pt as a cocatalyst. Ultimately, these strategies enabled the CZTS‐based photocathode to achieve excellent PEC performance under solar irradiation.^[^ ^42^, ^43^ ^]^ In 2015, we found that the modification of the In_2_S_3_/CdS overlayer onto the CZTS film can improve the stability of the CZTS‐based photocathode.^[^ ^44^ ^]^ In 2018, we replaced TiO_2_ with HfO_2_ as the protective layer, the photocurrent density of the CZTS‐based photocathode reached 12 mA cm^−2^ (0 V_RHE_), and the CZTS‐BiVO_4_ tandem cell presented an unbiased solar‐to‐hydrogen (STH) efficiency over 1% for the first time.^[^ ^45^ ^]^ In 2020, we improved the STH efficiency to a record of 3.17% by surface/interfacial modification of a HfO_2_/CdS/HfO_2_ sandwich buffer layer onto a CZTS photoelectrode.^[^ ^46^ ^]^ Although the unbaised STH efficiency of the CZTS‐based photoelectrode and CZTS‐BiVO_4_ tandem cell improvements every year, the achieved record STH efficiency remains low. The photoelectrochemical STH efficiency still has a large room to be improved.^[^ ^47^ ^]^ Recently, we found that the utilization of the solar thermal spectrum that cannot be absorbed by the CZTS and CZTS‐BiVO_4_ tandem system dramatically increased the STH efficiency of the CZTS‐BiVO_4_ tandem cell to an excited value over 8%.

**Figure 1:**
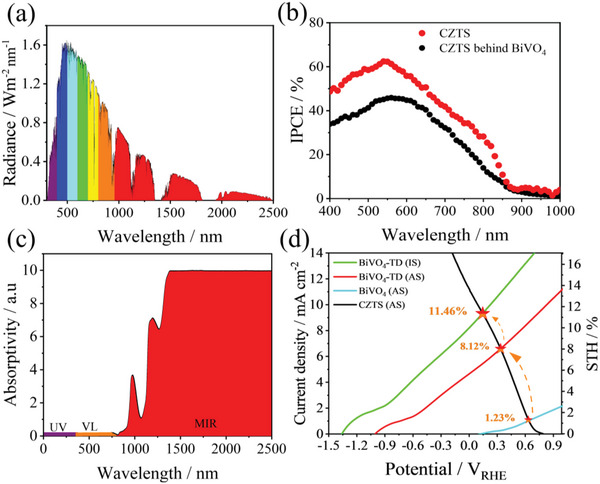
a) Radiance of the solar simulated AM 1.5 G; b) IPCE spectra of the CZTS‐based photocathode with and without filtering BiVO_4_ photoanode in tandem cell; c) absorbance of aqueous solution; d) comparison of STH efficiency between the actual state (AS) and ideal state (IS). Above measurements were carried out in 0.2 mol L^−1^ Na_2_HPO_4_/NaH_2_PO_4_ solution (pH 6.8) under solar simulated AM 1.5 G irradiation.

In the full spectrum of sunlight, ultraviolet (UV) light, visible light, and infrared light account for about 7%, 50%, and 43%, respectively. **Figure** **1**a shows the radiance of standard sunlight (AM 1.5 G), which clearly shows that near infrared (NIR) light occupies a high proportion of sunlight between 800 and 2500 nm. NIR has a strong thermal effect and accounts for ≈50% of the solar flux.^[^ ^48^ ^]^ Figure 1b shows the energy utilization efficiency of the CZTS film for different wavelengths of sunlight. We found that the effective absorption of sunlight by the CZTS‐based photocathode is mainly concentrated in the visible light band from 400 to 850 nm and the IPCE spectra always have long drop shoulder after about 550 nm, this phenomenon was caused by the defects from the bulk of CZTS and interface recombination at the heterointerface of CZTS/buffer.

**Figure 2:**
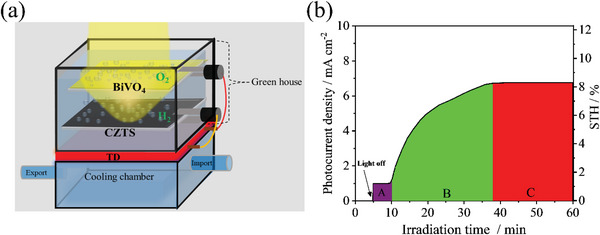
a) Schematic diagram of the TD‐Cu_2_ZnSnS_4_‐BiVO_4_ integrated tandem device; b) Light energy conversion efficiency cumulative curve.

Although infrared light accounts for ≈50% of solar radiation energy, infrared light is normally absorbed in the form of thermal energy rather than photons.^[^ ^49^ ^]^ Figure 1c shows the absorption efficiency of sunlight by aqua. It was found that infrared light can be absorbed by aqua, and aqua converts infrared light into thermal energy, resulting in an increase in aqua temperature. By an ideal design of a solar water splitting chamber such as the sealed green house (**Figure** **2**a), the increased temperature of the solution could be maintained and then utilized/recycled by integrated thermoelectric devices (TD) to improve the water splitting reactions. At the same time, we set up a cooling chamber to simulate the temperature of seawater to form an effective and stable temperature difference between the two ends of the thermoelectric device, so that the thermoelectric device can provide a continuous current to assist the photoelectrode to split water. It can also reduce the surface temperature of the CZTS and BiVO_4_ photoelectrodes to avoid any surface damage caused by solar thermal treatment. According to the law of conservation of energy and Joule's law without consideration of the heat loss, under 1 h of simulated illumination (AM 1.5 G), the temperature of the aqua could increase from 293 to 305 K (details are stated in the Supporting Information). The temperature difference of 12 K results in an additional bias of 1.0 V from the TD under the CZTS‐BiVO_4_ tandem cell. In this ideal case, the TD‐CZTS‐BiVO_4_ integrated device will present an STH efficiency over 11% (Figure 1d).

It should be noted here that the designed greenhouse device as shown in Figure 2a also acts as a solar thermal energy storage cell, greenhouse absorbed infrared solar light and storage as thermal energy with the solar light illumination time, while TD converted thermal energy into electric power, electric power was also be recycled and added onto CZTS‐BiVO_4_ tandem cell for enhanced overall water splitting. As shown in Figure 2b, CZTS‐BiVO_4_ tandem cell inside of the greenhouse presented simultaneous unbiased stable photocurrent density of about 1 mA cm^−2^ (about 1.2% STH efficiency) under solar light illumination during the initial 5–10 min illumination time (region A). After 10 min, TD was start to conversion the storage solar thermal energy of greenhouse into electric power that was also added onto CZTS‐BiVO_4_ tandem cell. Thus, the photocurrent density of CZTS‐BiVO_4_ tandem cell significantly increased from 1 mA cm^−2^ (1.2% STH efficiency) to about 6.7 mA cm^−2^ (8.12% STH efficiency). In this process (region B), the solar thermal energy storage speed was larger than the heat energy conversion rate by TD, dynamic solar thermal energy storage and conversion (by TD) inside of greenhouse with the solar light illumination time from 10 to 38 min was presented, the photocurrent density increased from the photocurrent density of CZTS‐BiVO_4_ tandem cell significantly increased from 1 mA cm^−2^ (1.2% STH efficiency) to about 6.7 mA cm^−2^ (8.12% STH efficiency). After 38 min light soaking (region C), the solar thermal energy storage and conversion (by TD) reach equilibrium, the photocurrent density and STH efficiency of CZTS‐BiVO_4_ tandem cell stabled at 6.7 mA cm^−2^ and 8.12%, respectively. Similar to various thin film solar cell, the device in this work also presented significant light soaking effect. STH efficiency of the device significantly enhanced with the light soaking time. It should be noted here that the 8.12% STH efficiency of the device is different from the previously reported transient STH efficiency of various devices.^[^ ^24^, ^25^, ^26^, ^27^, ^28^, ^29^, ^30^, ^31^, ^39^, ^40^, ^41^, ^42^, ^43^, ^44^, ^45^, ^46^ ^]^ The 8.12% STH efficiency of the device was achieved by solar light soaking over about 38 min, the 8.12% STH efficiency is more like the process accumulated STH efficiency. The STH efficiency discussed below in this work are all belong to the process accumulated STH efficiency. This is the first time to find the process accumulated STH efficiency and the light soaking effect of photoelectrode/device for solar water splitting.

Although the obtained STH efficiency of 8.12% is lower than the ideal value of over 11% because of some energy losses, the 8.12% STH efficiency has great industrial significance, while a large area (25 cm^2^) TD‐Cu_2_ZnSnS_4_‐BiVO_4_ integrated tandem device with superior long‐term stability was investigated for 1 week for the first time. This ingenious design greatly improves the STH efficiency of CZTS‐BiVO_4_ tandem cells by the synergistic coupling effect of solar thermal and PEC water splitting. This is the first report of a Cu_2_ZnSnS_4_‐BiVO_4_ tandem cell with a high STH efficiency of over 8% for solar water splitting. We will go further decrease the solar thermal loss by preparation of a sealed greenhouse chamber or using thermal protective coating inside of the chamber should decrease the solar thermal loss and increase the STH efficiency, and we believe that the STH efficiency of the TD‐CZTS‐BiVO_4_ integrated device will increase to a threshold over 10% with various efficient optimizations by the continuous efforts from the researchers among the world in the future.

In this study, a Pt‐HfO_2_/CdS/CZTS photocathode was prepared according to our previous research, and an applied bias photon‐to‐current efficiency (ABPE) of 2.8% and long‐term stability over 10 h were achieved. Moreover, a CZTS‐BiVO_4_ tandem cell assisted with a thermoelectric device (TD) was integrated with the CZTS‐BiVO_4_ tandem cell to utilize/recycle the solar thermal energy from infrared solar light (Figure 2a). We found that integration of a TD contributed an ≈1.0 V bias (≈12 K temperature difference) onto the CZTS‐BiVO_4_ tandem cell for solar water splitting. The voltage provided by TD significantly increases the photocurrent onset potential and photocurrent density (as shown in Figure 1d). In addition, this design could keep the buffer solution at a suitable temperature zone through the energy conversion (thermal energy to electrical energy) of the TD (Figure S1, Supporting Information), which effectively avoids any damage to the surface of the CZTS‐BiVO_4_ tandem cell by solar thermal treatment and maximize the use of solar energy. In addition, we also studied the effect of different temperatures on water splitting and analyze the reasons for the differences. It also further proves the importance of thermoelectric devices for improving the performance of photoelectrochemical (details are described in Figure S2, Supporting Information). The TD‐CZTS‐BiVO_4_ integrated device presented a remarkable solar‐to‐hydrogen efficiency (STH) of over 8%, which is a milestone value for CZTS‐based solar water splitting devices, especially for industrial utilization.

We performed the same test in natural seawater without any sacrificial reagents, achieving an STH efficiency of 2.46%. This is the first report to use a CZTS‐BiVO_4_ tandem cell for solar natural seawater splitting and it achieved an STH efficiency of 2.46%, which is also the highest value reported thus far. In addition, large‐area (25 cm^2^) TD‐Cu_2_ZnSnS_4_‐BiVO_4_ integrated tandem device was prepared and this large‐area device with superior long‐term stability was investigated for 1 week for the first time up to now. High STH efficiency and long‐term stability of TD‐Cu_2_ZnSnS_4_‐BiVO_4_ device indicated its great prospect in real applications.

## 2 Results and Discussion

### 2.1 The PEC Performance of CZTS‐Based Photocathodes

Referencing our previous work,^[^ ^40^, ^45^, ^46^ ^]^ CZTS thin films were prepared on a Mo substrate by the spray pyrolysis method, followed by annealing and vulcanization. We used X‐ray diffraction (XRD) and Raman analyses (Figure S3, Supporting Information) to research the structural characteristics of CZTS, the results show that CZTS has a good structure and no obvious secondary phase.

**Figure 3:**
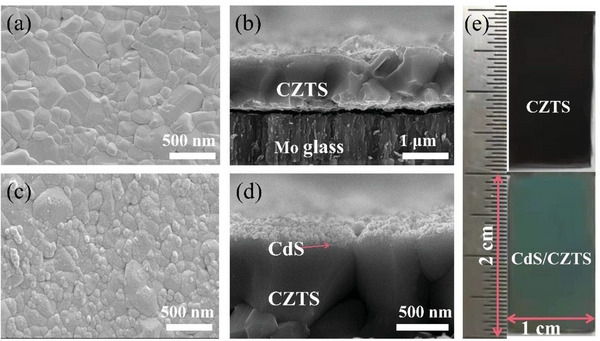
a) Surface and b) cross‐sectional SEM images of CZTS; c) surface and d) cross‐sectional SEM images of CdS/CZTS; e) morphology of CdS/CZTS in different preparation stages.

Panels a and b in **Figure** **3** show the surface scanning electron microscopy (SEM) image of CZTS, and the grains are densely covered on the molybdenum substrate to ≈1000 nm thickness. The surface of the well‐structured CZTS is brown‐black (Figure 3e). However, the photogenerated charge separation efficiency of the bare CZTS absorption layer is relatively low. We deposited an ≈200 nm thick N‐type CdS film on the surface of the CZTS film by the chemical bath deposition method (CBD) to form a P‐N junction (as shown in Figure 3c,d), which could effectively enhance the carrier separation efficiency. At this time, the surface of the CdS/CZTS appears light blue (Figure 3e). Compared with the bare CZTS film shown in Figure 3a, the surface of each CZTS grain is completely covered by small CdS particles. However, the photocorrosion of CdS seriously affects the PEC stability of CdS/CZTS‐based photocathodes.^[^ ^39^ ^]^ Based on our previous work, we modified the surface of CdS/CZTS with HfO_2_, and deposited an ≈6 nm thick HfO_2_ film on the CdS/CZTS film by atomic layer deposition (ALD).^[^ ^45^ ^]^ In order to further confirm whether CdS and HfO_2_ form an effective modification on the CZTS surface, we test the XPS spectra of the CZTS‐based photocathode surface (shown in Figure S4, Supporting Information), it was verified that the HfO_2_/CdS layer effectively modified the CZTS surface.

**Figure 4:**
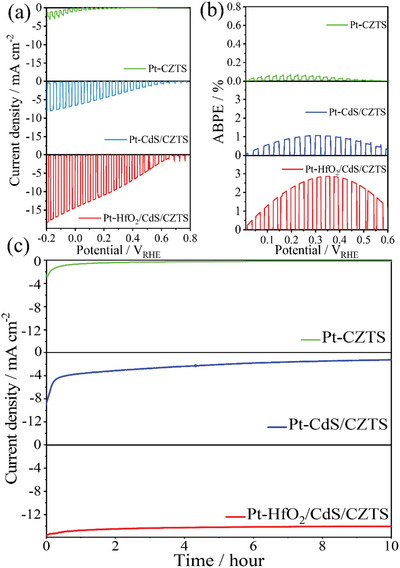
a) Chopped photocurrent density‐potential curves of (Pt‐CZTS, Pt‐CdS/CZTS, Pt‐HfO_2_/CdS/CZTS) photocathodes, b) its ABPE curves and c) photocurrent density–time curves. Above measurements were carried out in 0.2 mol L^−1^ Na_2_HPO_4_/NaH_2_PO_4_ solution (pH 6.8) under solar simulated AM 1.5 G irradiation.

**Figure** **4**a shows the photoelectric conversion efficiency of the bare CZTS absorption layer was extremely low, and the photocurrent density was only 2 mA cm^−2^ (0 V_RHE_). For the purpose of improving the photocurrent density, we deposited CdS on the surface of CZTS by the CBD method to form a heterojunction, which effectively improved photon absorption efficiency and incident photon conversion efficiency (Figure S5a, Supporting Information). At the same time, we deposited a layer of Pt ions on the surface of the CdS/CZTS photocathode to effectively improve the photocatalytic efficiency.^[^ ^50^ ^]^ The photocurrent density of Pt‐CdS/CZTS was increased to 7 mA cm^−2^ (0 V_RHE_), which is 3.5 times higher than that of bare CZTS, and the applied bias photon‐to‐current efficiency (ABPE) reached 1.07% (0.3 V_RHE_) as shown in Figure 4b. However, due to the photocorrosion of CdS, the photocurrent of Pt‐CdS/CZTS decreased rapidly. Figure 4c shows that the photocurrent density dropped to half of its initial value after 1 h. To improve the stability of the photoelectrode, we deposited an ≈6 nm thick HfO_2_ protective layer on the surface of CdS/CZTS by ALD. The HfO_2_ protective layer deposited on the CZTS effectively passivate the interface and inhibit the photoelectrode surface photocorrosion, which further reduced the resistance and improve photon absorption efficiency (shown in Figure S5b, Supporting Information). At this time, the photocurrent density reached 14 mA cm^−2^ (0 V_RHE_), and the ABPE reached 2.8% (0.35 V_RHE_), more importantly, the stability of the Pt‐HfO_2_/CdS/CZTS photocathodes was improved, which exceeding 10 h (shown in Figure 4c), 10 times higher than without HfO_2_ modification. This further illustrates that the HfO_2_ layer not only covers the CdS/CZTS grains but also passivates their grain boundaries, protecting the CdS/CZTS surface and greatly improved the carrier transfer rate and PEC stability of CZTS. In addition, the PEC performance of the photoelectrode was closely related to the external light intensity, which directly affects the efficiency of photoexcited carrier generation. We tested photocurrent density of Pt/HfO_2_/CdS/CZTS photoelectrode under different light intensity, when the light intensity increases from 50 to 200 mW cm^−2^, the photocurrent density of CZTS‐based photoelectrode at 0 V_RHE_ correspondingly increases from 10 to 22 mA cm^−2^ (shown in Figure S6, Supporting Information). The results show that the light intensity has a significantly effect the photocurrent density of the photoelectrode. The above research results provide a perfect foundation for subsequent work.

### 2.2 Performance Characterization of Thermoelectric Devices

**Figure 5:**
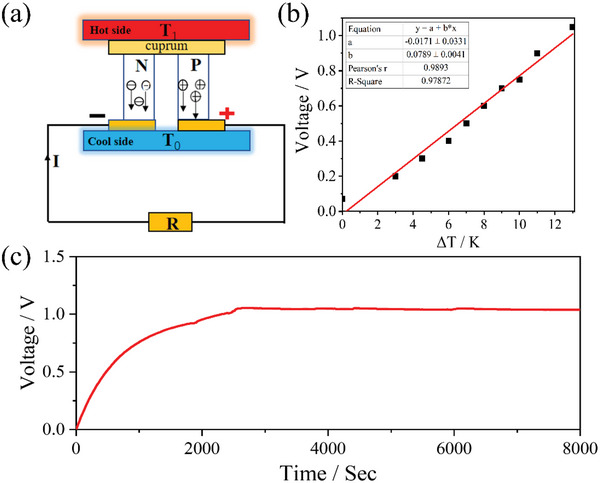
a) Principle of the TD; b) voltage‐temperature linear fitting curve of TD; c) thermoelectromotive force output curve of TD.

**Figure** **5**a shows the working principle of the thermoelectric device (TD). When there is a temperature difference (*T*_1_−*T*_0_) between the two sides of the P‐N junction, electrons will move from the hot side to the cold side, the thermoelectromotive force (*U*) is generated. Assuming *T*_1_ = *T*_0_ + ΔT, the thermoelectromotive force generated by Δ*T* is Φ_u_, calculate the derivative of *U* with respect to *T* could get the *α*, which is called Seebeck coefficient and the unit is V K^−1^. The relationship between *α*, *U*, and *T* accord with the following relation.

(1) $$\alpha =\frac{d\cup }{dT}$$

We use the two‐electrode method to test the open‐circuit voltage (details are stated in the Supporting Information) of the TD. Under the experimental conditions, we use recycled water as the cold source (room temperature at 293 K) to simulate the temperature of natural seawater (in fact, the average temperature of natural seawater is much lower than 293 K), while we use water pumps to form a circulating system to keep the temperature of the cooling chamber constant, it can provide constant temperature water (about 293 K) for the heat dissipation surface. When the circulating system is turned on, TD has the same temperature on both the cold and hot sides in the initial stage, both are equal to the room temperature, so the thermoelectromotive force is about 0 V at this time. As the green house absorbs the heat generated by the infrared light, the temperature of the hot side of TD gradually increases, while the temperature of the cooling chamber remains constant, and a thermoelectric potential is generated at this time. Figure 5c shows with the temperature difference between the two sides of the TD increases gradually, the TD system obtained a stable voltage of about 1.0 V after the first 2500 s, and remained stable for the subsequent period of time. Due to actual temperature changes, the voltage fluctuates within 70 mV. Ultimately, we extracted the key data and obtained the voltage‐temperature linear fitting curve of TD (shown in Figure 5b). The Seebeck coefficient of this TD is ≈0.08 V K^−1^ and the linear fitting curve also conforms to formula (1), which also illustrates the scientific of our experimental data. In this study, we tested the thermoelectric open circuit voltage more than 8000 s, this is the longest and most stable in this field so far. As long as the temperature difference between the two sides of TD is kept constant, the thermoelectromotive force can be continuously stable output.

### 2.3 The PEC Performance of TD‐CZTS‐BiVO4 Tandem Cell in Buffer Solution

**Figure 6:**
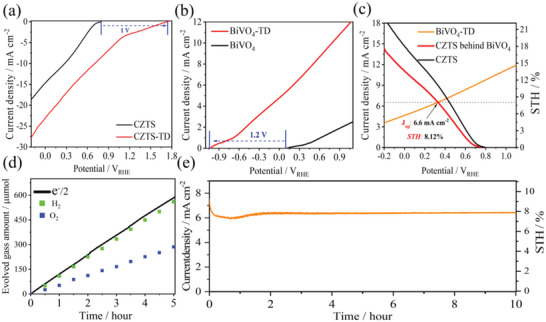
*J*–*V* curves of the a) CZTS‐based photocathode and b) BiVO_4_ photoanode with and without TD; *J*–*V* curves of the c) CZTS‐based photocathode, TD‐BiVO_4_ photoanode, and CZTS‐based photocathode behind the BiVO_4_ photoanode; d) hydrogen and oxygen evolution amount‐time curves (hydrogen and oxygen evolution produced from the tandem device detected by gas chromatography, the solid line denotes the time course curve of e^−^/2) of the TD‐CZTS‐BiVO_4_ tandem cell; e) photocurrent density‐time curve of the TD‐CZTS‐BiVO_4_ tandem cell. Above measurements were carried out in 0.2 mol L^−1^ Na_2_HPO_4_/NaH_2_PO_4_ solution (pH 6.8) under solar simulated AM 1.5 G irradiation.

In this study, we integrated thermoelectric devices (TD) with CZTS‐BiVO_4_ tandem cell to make full use of the entire solar spectrum. The photoexcited carrier transfer and solar hydrogen evolution reaction process is described in the band diagram (Figure S7, Supporting Information). **Figure** **6**a shows that without TD, the photocurrent onset potential (*V*_oc_) of the Pt‐HfO_2_/CdS/CZTS photocathode is ≈0.7 V_RHE_, and the photocurrent density (*J*_0_) is ≈14 mA cm^−2^ (0 V_RHE_). When we integrated the CZTS‐based photoelectrode with TD, the voltage provided from the TD enhanced the charge separation and transfer efficiencies of CZTS‐based photoelectrode, and the photocurrent onset potential (*V*_oc_) of the CZTS‐based photocathode was ≈1.7 V_RHE_. *V*_oc_ was advanced to ≈1 V_RHE_, and the obtained photocurrent density was ≈23 mA cm^−2^ (0 V_RHE_). *J*_0_ of TD‐CZTS exceeded 1.6 times that of CZTS and ABPE reached to 7.1%, which is an increase of ≈2.4‐fold (Figure S8a, Supporting Information). Similarly, Figure 6b shows that without TD, the *V*_oc_ of the BiVO_4_ photoanode was ≈0.2 V_RHE_, after integration with the TD, the *V*_oc_ of the TD‐BiVO_4_ photoanode was advanced to ≈1.2 V_RHE_, the photocurrent density (at 1.23 V_RHE_) was ≈5 times higher than that without TD, which increased from 2.5 to 12 mA cm^−2^. In order to further study the effect of TD on the charge separation process and energy band potential of BiVO_4_, we tested the EIS, OCP and M‐S of BiVO_4_ and TD‐BiVO_4_, respectively (shown in Figure S9, Supporting Information), indicating that the TD further optimized the PEC performance of BiVO_4_ and promotes water splitting efficiency of the CZTS‐BiVO_4_ tandem cell. Figure S8b (Supporting Information) shows that the BiVO_4_ photoanode and the CZTS‐based photocathode form a tandem cell, which has a low photocurrent without TD. At this time, the solar‐to‐hydrogen efficiency (STH) of CZTS‐BiVO_4_ was only ≈1.23%. In the 2‐h hydrogen production test, the average hydrogen production rate was 22 µmol h^−1^, Faraday efficiency also remains at 97% in the process of hydrogen production and the actual working current was ≈1 mA cm^−2^ (shown in Figure S8c,d, Supporting Information).

In theory, the thermodynamic potential required to split water into H_2_ and O_2_ is 1.23 V (Table S2, Supporting Information). However, due to the high overpotential on the surface of semiconductors, a higher potential (1.6–2.4 V) was required for the water splitting reaction. When we integrated TD with the CZTS‐BiVO_4_ tandem cell, the additional voltage provided by TD made the total voltage of the CZTS‐BiVO_4_ tandem cell far exceed the thermodynamic voltage requirements of solar water splitting, which greatly improves the energy utilization and photoelectric conversion efficiency. As a result, the TD‐CZTS‐BiVO_4_ integrated device presented a remarkable solar‐to‐hydrogen efficiency (STH) of over 8% under actual conditions (shown in Figure 6c). The STH efficiency was ≈7 times higher than that with no TD. As shown in Figure 6d, in the 5‐h hydrogen production test, the average hydrogen production rate was 112 µmol h^−1^, which was 5 times higher than that without TD and the Faraday efficiency remains at 97% during hydrogen production. This is the highest STH efficiency and the highest hydrogen production rate achieved in the field of Cu_2_ZnSnS_4_‐BiVO_4_ integrated tandem cells for solar water splitting thus far (**Table** **1**). Figure 6e demonstrates that the tandem cell integrated TD possessed a very high long‐term stability, and the photocurrent after 10 h of irradiation maintained ≈93% of its initial value. As shown in Figure S10 (Supporting Information), we also explored the TD‐driven electrocatalytic performance on the CZTS‐BiVO_4_ integrated tandem cell, it is found that photocurrent of the CZTS‐BiVO_4_ tandem cell under dark environment is 0 mA cm^−2^ regardless of integration of thermoelectric devices (TD), only when there is sunlight, the performance of TD‐driven electrocatalytic performances can be significantly improved. It further shows that the energy source of the TD‐CZTS‐BiVO_4_ or CZTS‐BiVO_4_ integrated tandem cell is all solar energy. We think that is a synergistic coupling effect of solar thermal and photoelectrochemical water splitting, only dependent on TD or CZTS‐BiVO_4_ tandem cell can't achieve appreciable STH efficiency. We also can expect further improvement in STH efficiency by system optimizations on TD and the TD‐CZTS‐BiVO_4_ tandem cell.

**Table 1:** Comparison of the photoelectrochemical performances of various typical tandem cells to date

Photoelectrodes	pH of buffer	*V*_oc_ [V_RHE_]	*J* [mA cm^−2^]; ABPE [%]; Durability [h] (% remain)	Tandem cell modules	Tandem cell STH and durability [h] (% remain) (in buffer solution)	Tandem cell STH and durability [h] (% remain) (in natural seawater)	Reference (year)
a. Highest STH achieved by CZTS photocathodes with any photoanode in tandem cell							
Pt‐HfO_2_/CdS/CZTS	6.8	1.7	−23(0 V_RHE_); 4.41%; 10 h (97%)	TD‐CZTS‐BiVO_4_	8.12%; 10 h (92%)	2.46%; 10 h (97%)	This work
Pt‐TiO_2_/CdS/CZTS	9.5	0.6	−9 (0 V_RHE_); 1.2%; NR	NR	NR	NR	Yokoyama et al. 2(010)^[^ ^41^ ^]^
Pt‐CdS/CZTS	9.0	0.45	−2.5 (0 V_RHE_); NR; NR	NR	NR	NR	Ma et al. (201)1^[^ ^42^ ^]^
Pt‐TiO_2_/AZO/CdS/CZTS	7.0	0.2	−1 (0 V_RHE_); NR; 0.25 h (55%)	NR	NR	NR	Rovelli et al. (2013)^[^ ^39^ ^]^
Pt‐In_2_S_3_/CdS/CZTS	6.5	0.6	−9.3 (0 V_RHE_); 1.63%; 3 h (100%)	CZTS‐BiVO_4_	0.4%; 2 h (80%)	NR	Jiang et al. (2015)^[^ ^44^ ^]^
Pt‐HfO_2_/CdS/CZTS	6.8	0.65	−12.8 (0 V_RHE_); 2.7%; 10 h (100%)	CZTS‐BiVO_4_	1.05%; 10 h (93%)	NR	Jiang et al. (2018)^[^ ^45^ ^]^
MoS_x_‐CdS/CZTS	6.5	0.6	−14 (0 V_RHE_); 3%; 10 h (70%)	NR	NR	NR	Feng et al. (2020)^[^ ^40^ ^]^
Pt‐HfO_2_/CdS/HfO_2_/CZTS	6.5	0.7	−18 (0 V_RHE_); 5.6%; 24 h (97%)	CZTS‐BiVO_4_	3.17%; 20 h (85%)	NR	Huang et al. (2021)^[^ ^46^ ^]^
b. Highest STH achieved by BiVO_4_ photoanode with any photocathodes in tandem or overall water splitting cell							
Pt‐HfO_2_/CdS/CZTS	6.8	1.7	−23(0 V_RHE_); 4.41%; 10 h (97%)	TD‐CZTS‐BiVO_4_	8.12%; 10 h (92%)	2.46%; 10 h (97%)	This work
RuOx‐Cu_2_O	6.0	0.55	−3.0 (0 V_RHE_); NR; NR	BiVO_4_‐Cu_2_O	1.23%; 2 min (40%)	NR	Bornoz et al. (2014)^[^ ^13^ ^]^
NiOOH/FeOOH/BiVO_4_	7.0	0.25	6.0 (1.23 V_RHE_); 2.4%; NR	BiVO_4_‐PSC‐Pt	6.2%; 10 h (94%)	NR	Qiu et al. (2016)^[^ ^18^ ^]^
Pt‐CdS/CuGa_3_Se_5_/GaSe_2_	7.0	0.8	−8 (0 V_RHE_); 1.4%; NR	BiVO_4_‐ACGSe	0.67% 2 h (99%)	NR	Kim et al. (2016)^[^ ^15^ ^]^
Pt‐CdS/CuIn_0.5_Ga_0.5_Se_2_	6.8	0.7	−28 (0 V_RHE_); 12.5%; NR	BiVO_4_‐CIGS	3.7%; 10 min (81%)	NR	Kobayashi et al. (2018)^[^ ^24^ ^]^
RuOx‐TiO_2_/Ga_2_O_3_/Cu_2_O	5.0	1.0	−10 (0 V_RHE_); NR; 120 h (80%)	BiVO_4_‐Cu_2_O	3%; 12 h (90%)	NR	Pan et al. (2018)^[^ ^25^ ^]^
Perovskite/Field's metal‐Pt	8.5	0.8	−12 (0 V_RHE_); NR; 7 (75%)	BiVO_4_‐Perovskite	0.35% 18 h(71%)	NR	Andrei et al. (2018)^[^ ^28^ ^]^
Pt‐TiO_2_/CdS/Sb_2_Se_3_	1.0	0.5	−20 (0 V_RHE_); 3.4%; NR	BiVO_4_‐Sb_2_Se_3_	1.5%; 10 h (90%)	NR	Yang et al. (2020)^[^ ^26^ ^]^
FTO/WO_3_/BiVO_4_	7.0	0.3	3.0 (1.23 V_RHE_); NR; NR	BiVO_4_‐GaAs_1‐x_P_x_	2%; 3 h (52%)	NR	Lawrence et al. (2021)^[^ ^17^ ^]^
Pt‐C_60_pat‐TiO_2_ball/GeSe	6.8	0.6	−11 (0 V_RHE_); 1.14%; 20 h (88%)	BiVO_4_‐GeSe	1.37%; 12 h (88%)	NR	Wang et al. (2021)^[^ ^30^ ^]^
Pt‐TiO_2_/CdS/Cu_3_BiS_3_	6.5	0.9	−7 (0 V_RHE_); 1.7%; 10 h (90%)	BiVO_4_‐Cu_3_BiS_3_	2.04%; 20 h (90%)	NR	Huang et al. (202)1^[^ ^31^ ^]^
FeCoO_x_/BiVO_4_	6.0	0.4	−0.6 (1.23 V_RHE_); NR; NR	BiVO_4_‐ZrO_2_/TaON	0.6%; NR	NR	Domen et al. (2022)^[^ ^12^ ^]^
WO_3_/BiVO_4_/Bi_2_S_3_	10.8	0.2	1.5 (1.23 V_RHE_); 0.78%; 6 h (90%)	Pt‐BiVO_4_	NR	NR	Jeganathan et al. (2022)^[^ ^19^ ^]^
BVO‐ZCIS_DDT_	9.0	0.2	3.8 (1.23 V_RHE_); 0.9%; NR	BVO‐Cu_2_O‐ZCIS_DDT_	0.65%; 2 h (90%)	NR	Cai et al. (2022)^[^ ^21^ ^]^
Mo‐BiVO_4_	7.0	0.3	5.6 (1.23 V_RHE_); NR; NR	BiVO_4_‐SrTiO_3_	0.012%; 40 h (80%)	NR	Ma et al. (2022)^[^ ^23^ ^]^
Au/Ni/ITO/BiVO_4_@CoPi	7.0	0.2	5.2 (1.23 V_RHE_); NR; NR	PV‐Pt‐BiVO_4_	5.3%; 1.4 h (90%)	NR	Yang et al. (2022)^[^ ^51^ ^]^
c. Highest STH achieved by any PEC tandem cell using seawater							
Pt‐HfO_2_/CdS/CZTS	6.8	1.7	−23(0 V_RHE_); 4.41%; 10 h (97%)	TD‐CZTS‐BiVO_4_	8.12%; 10 h (92%)	2.46%; 10 h (97%)	This work
FTO/BiVO_4_/Mo‐RhO_2_	8.0	−0.2	−2.16 (1.0 V_RHE_); NR; 5 h (93%)	BiVO_4_‐Pt (bias of 1.2 V)	NR	6 µmol h^−1^ (evolution H_2_ rate)	Luo et al. (2011)^[^ ^52^ ^]^
Si/p‐GaN/InGaN	8.0	NR	NR	NR	NR	1.9%; NR	Guan et al. (2018)^[^ ^53^ ^]^
COP‐TF@CNi_2_P	12	NR	NR	NR	NR	2.5 mol g^−1^ h^−1^; 48 h (92%)	Liu et al. (2019)^[^ ^54^ ^]^
Pt/GaP‐TiO_2_‐SiO_2_	8.0	NR	NR	NR	NR	41.5 µmol g^−1^ h^−1^; 4 h (NR)	Dang et al. (2021)^[^ ^55^ ^]^
H‐CoS/CdS‐2	7.0	NR	NR	NR	NR	143 µmol g^−1^ h^−1^; 4 h (NR)	Liu et al. (2022)^[^ ^56^ ^]^

*J*, photocurrent density; *V*_oc_, the photocurrent onset potential; ABPE, applied bias photon‐to‐current efficiency; STH, solar to hydrogen; TD, thermoelectric device; NR, not reported; PV, photovoltaic solar cell.

### 2.4 The PEC Performance of TD‐CZTS‐BiVO_4_ Tandem Cell in Natural Seawater

**Figure 7:**
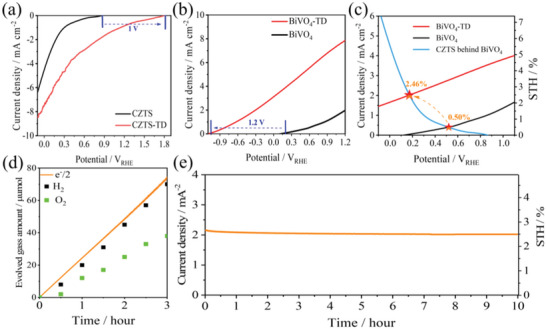
*J*–*V* curves of the a) CZTS‐based photocathode and b) BiVO_4_ photoanode with and without TD; *J*–*V* curves of the c) BiVO_4_ photocathode, TD‐BiVO_4_ photoanode, and CZTS‐based photocathode behind the BiVO_4_ photoanode; d) hydrogen and oxygen evolution amount‐time curves (hydrogen and oxygen evolution produced from the tandem device detected by gas chromatography, the solid line denotes the time course curve of e^−^/2) of the TD‐CZTS‐BiVO_4_ tandem cell; e) photocurrent density‐time curve of the TD‐CZTS‐BiVO_4_ tandem cell. Above measurements were carried out in natural seawater (pH 7.5) under solar simulated AM 1.5 G irradiation.

**Figure 8:**
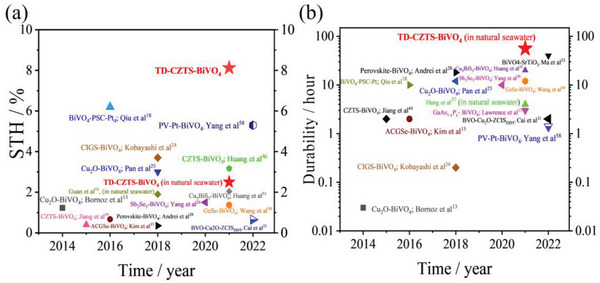
Efficiency benchmarks of the previously reported photocathode–photoanode tandem cells for solar water splitting. a) STH efficiency and b) durability benchmarks of the reported photocathode–photoanode tandem cells for standalone water splitting in recent years. (★: this work).

Considering that the TD‐CZTS‐BiVO_4_ integrated tandem cell will be applied to split seawater in the future, we performed the same test in natural seawater (Estuary of the Pearl River, Guangzhou on May 3, 2021). The main chemical composition in natural seawater are Na^+^, K^+^, Ca^2+^, Mg^2+^, Sr^2+^ Cl^−^, Br^−^, F^−^, CO_3_^2−^, and SO_4_^2−^. These ions are mainly present in the form of salt ions, which accounts for more than 99% (chlorine and sodium make up nearly 90%) of the total chemical elements in natural seawater. In this study, as shown in Table S3 (Supporting Information), we tested some major ion concentrations (ion concentration exceeds 100 mg L^−1^) in natural seawater based on research needs and compared it with the buffer solution. **Figure** **7**a shows the photocurrent onset potential (*V*_oc_) of the Pt‐HfO_2_/CdS/CZTS photocathode is ≈0.7 V_RHE_, and the photocurrent density (*J*_0_) is ≈3.5 mA cm^−2^ (0 V_RHE_). When we integrated the CZTS‐based photoelectrode with TD, the photocurrent onset potential (*V*_oc_) of the TD‐CZTS was ≈1.7 V_RHE_. *V*_oc_ was advanced to ≈1 V_RHE_, and the obtained photocurrent density was ≈7 mA cm^−2^ (0 V_RHE_). The *J*_0_ and ABPE of the TD‐CZTS are ≈2 times and 5 times higher than that of the CZTS‐based photoelectrode, respectively (Figure S11a, Supporting Information). Similarly, the *V*_oc_ of the BiVO_4_ photoanode was increased from the initial value of about 0.2 V_RHE_ to ≈−1.2 V_RHE_ when we integrate TD onto BiVO_4_ (Figure 7b, ), the CZTS‐based photocathode and the BiVO_4_ photoanode showed similar change trends as that in the 0.2 mol L^−1^ Na_2_HPO_4_/NaH_2_PO_4_ solution, however, the photocurrent density decreased compared to the buffer solution. The main reason for the difference is that the concentration of ions in the solution is different, it is worth noting that the ion concentration in natural seawater is much lower than the ion concentration in the 0.2 mol L^−1^ Na_2_HPO_4_/NaH_2_PO_4_ solution (shown in Table S3, Supporting Information). The ion concentration is closely related to the conductivity of the solution, which in turn leads to a decrease in the photocurrent density in seawater compared to the buffer solution.In addition, H^+^ ion in buffer solution is also an important reason for the increase of photocurrent density.^[^ ^40^, ^46^ ^]^ As shown in Figure S12 (Supporting Information), we tested the influence of ion concentration and temperature on the conductivity of the solution respectively. The results show that the ionic concentration has a significant effect on the conductivity of the solution compared with the solution temperature (Figure S12a, Supporting Information), in addition, the temperature of the solution also has a slight effect on the conductivity of the solution (Figure S12b, Supporting Information) and the conductivity of the solution corresponds to the change in photocurrent. When there is no TD, the working current density of the Cu_2_ZnSnS_4_‐BiVO_4_ tandem cell in seawater is only ≈0.41 mA cm^−2^, and the STH efficiency is only 0.50% (shown in Figure 7c). When we integrated the Cu_2_ZnSnS_4_‐BiVO_4_ tandem cell with TD, the working current density and STH efficiency of the TD‐CZTS‐BiVO_4_ integrated tandem cell reached 2.0 mA cm^−2^ and 2.46%, respectively. Compared with no TD, it was increased by ≈5 times. As shown in Figure 7d, the hydrogen production rate in the 3‐h test also reached the current highest 24 µmol h^−1^ when with TD in natural seawater and the Faraday efficiency reached 97%. Compared with CZTS‐BiVO_4_ tandem cell without TD (Figure S11b, Supporting Information), the hydrogen production rate is only about 5 µmol h^−1^ and the Faradaic efficiency is about 95%. It can be seen that the TD significantly improves the hydrogen production efficiency. Figure 7e demonstrates that the Cu_2_ZnSnS_4_‐BiVO_4_ tandem cell integrated with TD possessed a very high long‐term stability in natural pure seawater, and the photocurrent after 10 h of irradiation maintained ≈96% of its initial value. This is the first report to use sunlight to split natural seawater and the achieved unbiased STH efficiency of 2.46% is very competitive among the reported STH efficiencies of typical tandem cells (**Figure** **8** and Table 1).

**Figure 9:**
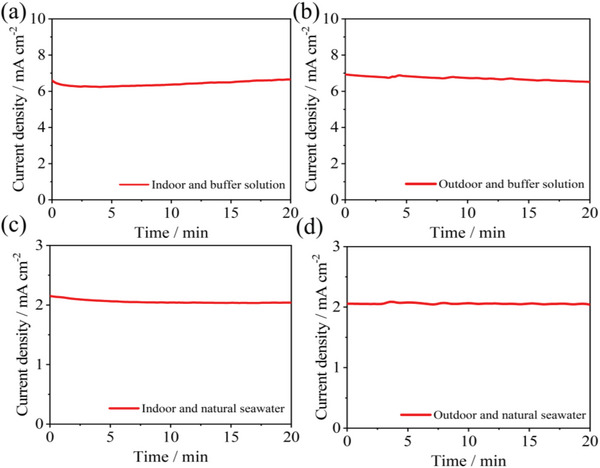
Comparison of conductivity between buffer solution (0.2 mol L^−1^ Na_2_HPO_4_/NaH_2_PO_4_ solution; pH 6.8) and natural seawater (pH 7.5), a) indoor and buffer solution, b) outdoor and buffer solution, c) indoor and natural seawater, d) outdoor and natural seawater.

Simultaneously, to study the difference between natural conditions and laboratory conditions, we tested the photocurrent of the TD‐CZTS‐BiVO_4_ tandem cell in different environments (shown in **Figure** **9**), In the buffer solution, the photocurrent of the TD‐CZTS‐BiVO_4_ tandem cell was kept around 6.5 mA cm^−2^ indoors and outdoors (Figure 9a,b). In natural seawater, it is also maintained at around 2 mA cm^−2^ regardless of whether it was indoors or outdoors (Figure 9c,d), which proved that almost no change in PEC performance of TD‐CZTS‐BiVO_4_ tandem cell in the same solution even with different environments. This further proves that this study has actual application value. Our design has unique advantages in both STH efficiency (2.46%) and stability, which has stronger practical application value, for example, the solar seawater splitting for hydrogen evolution.

### 2.5 Industrial Feasibility Study of Large‐Area TD‐CZTS‐BiVO_4_ Integrated Device

**Figure 10:**
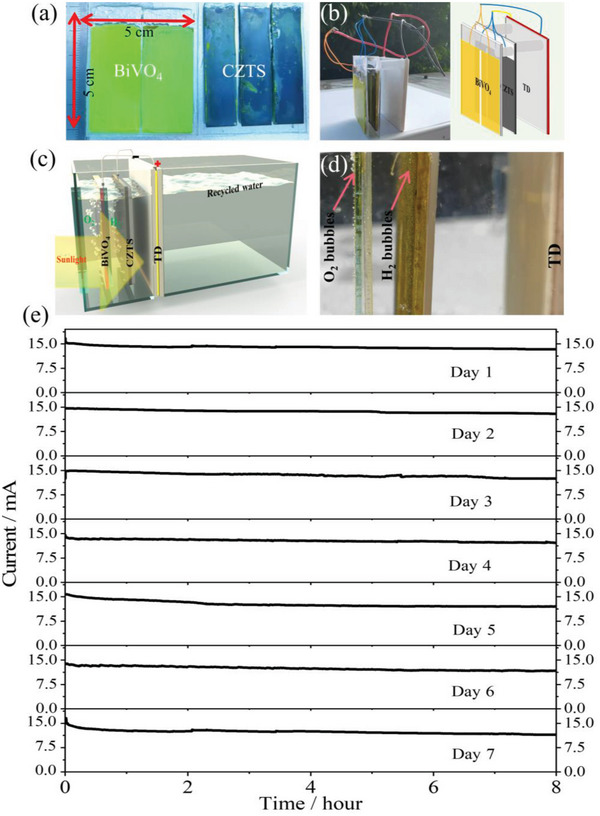
Photographs of a) 5 × 5 cm integrated CZTS‐based photocathode, 5 × 5 cm BiVO_4_ photoanode; b) large area (5 × 5 cm) integrated TD‐CZTS‐BiVO_4_ tandem device and diagram of large area (5 × 5 cm) integrated TD‐CZTS‐BiVO_4_ tandem device; c) schematic diagram of the photoelectrode integrated with TD; d) working photographs of the large area (5 × 5 cm) integrated TD‐CZTS‐BiVO_4_ tandem device under outside natural sunlight irradiation; e) photocurrent‐time curve of the large area (5 × 5 cm) integrated TD‐CZTS‐BiVO_4_ tandem device.

As we know, the reported photoelectrode devices including CZTS for solar water splitting are always in a small area device (less than 1 cm^2^).^[^ ^38^, ^40^, ^44^, ^45^, ^51^, ^52^, ^53^, ^54^, ^55^, ^56^, ^57^ ^]^ In this work, we prepared a 5 × 5 cm large tandem cell integration of Pt‐HfO_2_/CdS/CZTS photocathode and BiVO_4_ photoanode (**Figure** **10**a). We integrated a large‐area (25 cm^2^) hydrogen‐producing device based on TD‐CZTS‐BiVO_4_ (Figure 10b) for the first time, and evaluated the application potential of solar natural seawater splitting performances of TD‐CZTS‐BiVO_4_ integrated device (Figure 10c). Simultaneously, we tested the actual hydrogen production efficiency of the TD‐CZTS‐BiVO_4_ tandem device in different experimental environments, Videos S1 and S2 (Supporting Information) show the hydrogen production performances of the large area (25 cm^2^) TD‐CZTS‐BiVO_4_ integrated tandem cell in 0.2 mol L^−1^ Na_2_HPO_4_/NaH_2_PO_4_ solution and natural seawater under AM 1.5 G simulate sunlight irradiation, respectively. It can be observed that significant amount hydrogen and oxygen bubbles produced at the surface of the 25 cm^2^ TD‐CZTS‐BiVO_4_ tandem device in both the buffer solution and natural pure seawater under simulated solar light. In order to evaluate the real application potential at outside environment, we performed the solar hydrogen properties of the tandem device at outside for natural sunlight irradiation. Videos S3 and S4 (Supporting Information) show the solar hydrogen production performances from the large area (25 cm^2^) TD‐CZTS‐BiVO_4_ integrated tandem cell in 0.2 mol L^−1^ Na_2_HPO_4_/NaH_2_PO_4_ solution and natural seawater under natural sunlight outside (Sunny, 11:00 AM, July 7th, Guangzhou, China), respectively. It was found that the 25 cm^2^ TD‐CZTS‐BiVO_4_ tandem device was worked well under outside natural sunlight in both the buffer solution and natural pure seawater. Figure 10d and Figure S13 (Supporting Information) show the momentary actual working status of the large area (25 cm^2^) TD‐CZTS‐BiVO_4_ integrated tandem cell under outside natural sunlight irradiation in pure natural seawater, which clearly demonstrates dense bubbles on the surface of the photoelectrode. Furthermore, long time stability or reproducibility of the large area (25 cm^2^) tandem device is another key point for real application. As shown in Figure 10e, the large area (25 cm^2^) TD‐Cu_2_ZnSnS_4_‐BiVO_4_ integrated tandem device with superior long‐term stability was investigated for one week in this work for the first. We have tested for 8 h one day (simulate the duration of natural sunlight from 9:00 AM to 17:00 PM in one day) for seven consecutive days. It was found that the TD‐CZTS‐BiVO_4_ tandem device presented very high stability and reproducibility in one week, which indicates that the design of the CZTS‐BiVO_4_ tandem cell integrated TD has unique advantages and great development potential in terms of solar seawater splitting to produce hydrogen and oxygen and a huge application prospect in the industrial field, as well as protecting the environment and saving energy.

## 3 Conclusion

In this study, light soaking effect and process accumulated STH efficiency were first discovered for CZTS based solar water splitting photoelectrode. A record of process accumulated STH efficiency of 8.12% for the CZTS‐BiVO_4_ tandem cell was achieved by the synergistic coupling effect of solar thermal and photoelectrochemistry. This is the first report of a Cu_2_ZnSnS_4_‐BiVO_4_ tandem cell with a high unbiased STH efficiency of over 8% for solar water splitting due to the greenhouse device system design. The greenhouse acts as a solar thermal energy storage cell, which absorbed infrared solar light and storage as thermal energy with the solar light illumination time, while TD converted thermal energy into electric power, electric power was also be recycled and added onto CZTS‐BiVO_4_ tandem cell for enhanced overall water splitting. To utilize the solar thermal spectrum, a thermoelectric device (TD) was integrated with the CZTS‐BiVO_4_ tandem cell to utilize the wasted solar thermal energy generated from infrared solar light. It was found that the TD not only produced a significant additional thermoelectric bias of ≈1.0 V on the CZTS‐BiVO_4_ tandem cell to contribute to solar water splitting reactions but also reduced the surface temperature of the CZTS and BiVO_4_ photoelectrodes to avoid any surface damage caused by solar thermal treatment. Furthermore, for the first time, we have shown the solar natural seawater splitting ability of the TD‐CZTS‐BiVO_4_ integrated tandem cell. A champion STH efficiency of 2.46% of the TD‐CZTS‐BiVO_4_ integrated tandem cell in natural seawater was also first reported, while the large area (25 cm^2^) TD‐Cu_2_ZnSnS_4_‐BiVO_4_ integrated tandem device with superior long‐term stability was investigated for one week in this work for the first time up to now. The integration of a thermoelectric assistance system onto a CZTS‐BiVO_4_ tandem cell significantly enhanced the STH conversion efficiency to an applicable milestone, giving new insight into photoelectrochemical solar water splitting devices and providing a practical road to industrialization and large applications.

## 4 Experimental Section

### Preparations of CZTS Films

Spray pyrolysis method was used to prepare Cu_2_ZnSnS_4_ thin film on Mo‐substrate. The precursor solution was mixed by equal volume aqueous solution of Cu(NO_3_)_2_ (17 mm), Zn(NO_3_)_2_ (11.5 mm), Sn(CH_3_SO_3_)_2_ (11.5 mm), and thiourea SC(NH_2_)_2_ (50 mm). Firstly, the Cu‐Zn stock solution was formed by 0.49 g Cu(NO_3_)_2_·3H_2_O and 0.41 g Zn(NO_3_)_2_·6H_2_O both dissolved in 40 mL pure water. Subsequently, the TU stock solution was formed by 0.46 g SC(NH_2_)_2_ dissolved in 40 mL pure water. The Sn stock solution was formed by 550 µL Sn(CH_3_SO_3_)_2_ solution (50 wt%) dissolved in 40 mL pure water, and the pH of this stock solution was adjusted to 1.5 by adding 60 µL HNO_3_ These three stock solutions were all separately stirred for at least 2 h before their mix. Then 6 mL of above three stock solutions successively were taken to stir and mix, the mixed solution was immediately sprayed onto a cleaned Mo‐substrate which preheated to about 683 K, the CZTS thin film was densely covered on Mo‐substrate to ≈1000 nm thickness in 10 min. At last, the CZTS thin film was vacuum sealed in a glass tube with 20 mg sulfur powder and subjected to sulfurization in a furnace at 873 K for 30 min. All these reagents were of analytical grade and used without any purification in this experiment. The water solution used in the experiment was prepared using deionized water

### Surface Modification with CdS

A CdS layer was deposited onto the CZTS overlayer by the chemical bath deposition method (CBD). The prepared CZTS overlayer was dipped into an aqueous solution containing 12.5 mm CdSO_4_, 0.22 mm SC(NH_2_)_2_, and 11 m NH_4_OH at 333 K for 15 min.

### Surface Passivation with HfO_2_

An ultrathin HfO_2_ layer was deposited onto the CZTS films by atomic layer deposition (ALD) method. HfO_2_ was grown by using hafnium source and H_2_O as oxygen source. The growth rate was estimated to be about 0.1 nm per cycle and the films were grown for 60 cycles at 523 K. A HfO_2_ passivation layer was formed by depositing again a 60 ALD‐cycles HfO_2_ (6 nm) layer onto CdS/CZTS overlayer.

### Surface Modification with Photoelectron Deposition Pt Catalyst

The Pt particles deposition was performed by using a three‐electrode system consisting of HfO_2_/CdS/CZTS as a working electrode, a Pt wire as a counter electrode, and Ag/AgCl as a reference electrode. These electrodes were put in 0.1 m Na_2_SO_4_ solution containing 1 mm H_2_PtCl_6_, and the deposition was performed with a constant potential of −0.1 V_VS. Ag/AgCl_ by using CHI660E electrochemical measurement unit, the duration of deposition was 60 s. During the deposition process, the working electrode was illuminated by simulated AM 1.5 G solar irradiation.

Finally, a photocathode based on Pt‐HfO_2_/CdS/CZTS was formed.

### Preparation of BiVO_4_ Photoanode

There were three main steps in the preparation of BiVO_4_. Firstly, BiOI was prepared by electrodeposition method, then BiVO_4_ was obtained by annealing, and finally Co‐Pi catalyst was deposited on the surface of BiVO_4_ by photoelectric deposition method. The preparation details of the BiVO_4_ samples could be reference to previous works by Kuang et al.^[^ ^58^ ^]^ and Krol et al.^[^ ^59^ ^]^

### The Structure of TD and TD‐CZTS‐BiVO_4_ Hydrogen Production Cell

The thermoelectric device (TD) auxiliary system consists of a commercial thermoelectric (Hubei Saigerui New Energy Technology Co., Ltd, Model:TEG1‐19913, size: 50 mm × 50 mm × 4 mm, AC internal resistance (Ω.300 k) 1.58, Particle material: BiTe). BiTe is a semiconductor material with thermoelectric effect, when there is a temperature difference on the surface of the thermoelectric device, the electrons inside the BiTe flow from the high temperature end to the low temperature end, thereby generating a thermoelectric voltage. At the same time, the thermoelectric device with the photoelectrode through a reasonable package design to maximize the use of the energy of sunlight and improve the STH efficiency is integrated.

The structure of TD‐CZTS‐BiVO_4_ hydrogen production cell is mainly composed of a green room and a cooling room. The CZTS‐BiVO_4_ tandem cell is located in the green room, and the TD is located between the green room and the cooling room. Part of the visible light is absorbed by the CZTS‐BiVO_4_ tandem cell to produce oxygen and hydrogen. The temperature of the aqueous solution in the green room increases under the irradiation of visible light and infrared light that are not absorbed by the CZTS‐BiVO_4_ tandem cell, and the generated heat energy is absorbed by the TD and converted into electrical energy to driver the CZTS‐BiVO_4_ tandem cell to produce hydrogen, and maximize the use of the full spectrum of sunlight. In the experiment, the negative electrode of the TD is connected to the CZTS based photocathode, and the positive electrode is connected to the BiVO_4_ based photoanode. This design not only greatly improves the conversion efficiency of solar energy to hydrogen, but also has a simple structure and excellent stability, which can meet the practical industrial application.

### Photoelectrochemical Measurements

CHI660E electrochemical measurement unit was used to characterize the main photoelectrode PEC, including photocurrent, photovoltage, Nyquist plots, etc. An online gas chromatography system (Shimadazu GC‐2014 gas analyzer equipped with a MS‐5A column and a thermal conductivity detector) was used to detect H_2_ and O_2_ during the PEC water splitting. Plasma emission spectrometer (Manufacturer, Spike Germany; model; SPECTRO ARCOS MV) was used to test and analyze the main ion concentration in natural seawater and buffer and a conductivity meter was used (Manufacturer, Shanghai INESA Scientific Instrument Co. Ltd. model, DDSJ‐308F) to test the conductivity of different solutions. Natural seawater used in the experiment was taken from the estuary of the Pearl River in Nansha Park, Guangzhou on May 3, 2021. The simulated sunlight AM 1.5 G from a 300 W Xenon lamp solar simulator was 100 mW cm^−2^.

Potentials referred to the Ag/AgCl electrode were converted to reversible hydrogen electrode using the Nernst equation:

(2) $$V_{\mathrm{RHE}}=V_{\mathrm{Ag}/\mathrm{AgCl}}+0.059\times \mathrm{pH}+0.199$$

Applied bias photon‐to‐current efficiency (ABPE) was determined from the current density‐potential response of the photocathodes by using the following equation:^[^ ^11^ ^]^

(3) $$\mathrm{ABPE}\left(\%\right)=\frac{J\times V\times 100}{P}$$

where *J* is the photocurrent density (mA cm^−2^), *V* is the applied potential (V_RHE_), and *P* is the intensity of simulated sunlight (100 mW cm^−2^).

Solar to hydrogen efficiency (STH) refers to PEC under light (AM 1.5 G). The total energy conversion efficiency is the proportion of the chemical energy produced in the solar energy input.

STH efficiency is calculated according to the following formula:

(4) $$\mathrm{STH}\left(\%\right)=\frac{J_{\mathrm{OP}}\times 1.23\times 100}{P}$$

where *J*_OP_ (mA cm^−2^) is the actual current density of Cu_2_ZnSnS_4_‐BiVO_4_ integrated tandem cell, and P (100 mW cm^−2^) is the intensity of simulated sunlight.

Faraday efficiency (*ɳ*_F_) refers to the utilization efficiency of actual electrons in the process of photoelectrochemical (PEC) water splitting, which is calculated according to the following formula:

(5) $$\eta_{F}\left(\%\right)=\frac{2m\times N_{A}}{Q}$$

where *m* is the molar amount of hydrogen produced during the calculation period, *N*_A_ is Avogadro's constant, *Q* is the total charge passing through the circuit during this period.

Incident photon to current efficiency (IPCE) can be calculated using the following equation:

(6) $$\mathrm{IPCE}=\frac{J\times 1240}{\lambda \times P_{\lambda }}$$

*J* refers to the photocurrent density (mA cm^−2^) obtained from the electrochemical workstation. *λ* and *P*_*λ*_ are the incident light wavelength (nm) and the power density obtained at the specific wavelength (mW cm^−2^), respectively.

Quantum law of photons (*E*) represents the relationship between the wavelength and energy of a photon, which is obtained according to the wave‐particle duality as follows

(7) $$E=\frac{hc}{\lambda }$$

where *h* is Planck's constant, *c* is the speed of light, and *λ* is the incident light wavelength.

### Structural Characterization

Crystalline structures of the CZTS‐based photocathode were determined by X‐ray diffraction (XRD), Raman spectroscopy and using a Rigaku Mini Flex X‐ray diffractometer, and a Jasco NRC 3100 laser Raman spectrophotometer. Surface and bulk chemical structures of photoelectrodes were examined by X‐ray photoelectron spectroscopy (XPS) using a Shimadzu AXIS ULTRA X‐ray photo‐electron spectrometer. Surface and cross‐section morphology were observed by scanning electron microscope (SEM) using Hitachi S‐4800 microscope.

## Conflict of Interest

The authors declare no conflict of interest.

## Author Contributions

F.J. conceived the content and idea of this work, analyzed the data, and wrote the paper. H.C. did the main experiments and characterizations of the CZTS‐based photocathode and the TD‐CZTS‐BiVO_4_ tandem device. W.Z., G.X., and Y.H. participated into some experiments of the preparation of the CZTS and BiVO_4_. X.W. and H.N. did some photoelectrochemical characterizations. S.I., Y.N., J.T., and L.Z. provided some consultations.

## Supporting information

Supporting Information

Supplemental Video 1

Supplemental Video 2

Supplemental Video 3

Supplemental Video 4

## Data Availability

The data that support the findings of this study are available from the corresponding author upon reasonable request.
