# Supplementary material for: Process Accumulated 8% Efficient Cu2ZnSnS4‐BiVO4 Tandem Cell for Solar Hydrogen Evolution with the Dynamic Balance of Solar Energy Storage and Conversion
Source: Adv Sci (Weinh). 2022 Dec 20;10(5):2205726. doi: 10.1002/advs.202205726 (PMC9929259; doi:10.1002/advs.202205726)
Supplement: Supplementary file 1 — Supporting Information [file ADVS-10-2205726-s004.pdf]

ADVANCED SCIENCE
Open Access

Supporting Information

for *Adv. Sci.*, DOI 10.1002/advs.202205726

Process Accumulated 8% Efficient Cu_2ZnSnS_4-$BiVO_4$ Tandem Cell for Solar Hydrogen Evolution with the Dynamic Balance of Solar Energy Storage and Conversion

Hongwei Cai, Weidong Zhao, Guohong Xiao, Yucheng Hu, Xiaomin Wu, Huanyang Ni, Shigeru Ikeda, Yunhau Ng, Jiahua Tao, Lingzhi Zhao* and Feng Jiang**

Process accumulated 8% efficient Cu_2ZnSnS_4-$BiVO_4$ tandem cell for solar hydrogen evolution with the dynamic balance of solar energy storage and conversion

Hongwei Cai[a], Weidong Zhao[a], Guohong Xiao[a], Yucheng Hu[a], Xiaomin Wu[a], Huanyang Ni[a], Shigeru Ikeda[e], Yunhau Ng[f], Jiahua Tao[d*], Lingzhi Zhao[a*] and Feng Jiang[a b c]*

[a] Institute of Hydrogen Energy for Carbon Peaking and Carbon Neutralization, School of Semiconductor Science and Technology, South China Normal University, Foshan 528225, China

[b] Donghai Laboratory, Zhoushan Zhejiang 316021, China

[c] Chengfeng Light Energy Science and Technology (Guangzhou) Limited Company, Huangpu District, Guangzhou, China

[d] Key Laboratory of Polar Materials and Devices, Ministry of Education, East China Normal University, Information Building, 500 Dongchuan Road, Shanghai 200241.

[e] Department of Chemistry, Konan University, 9-1 Okamoto, Higashinada, Kobe, Hyogo 658-8501, Japan

[f] School of Energy and Environment, City University of Hong Kong, Kowloon, Hong Kong 999077, China

*E-mail: fengjiang@m.scnu.edu.cn (F. Jiang); lzzhao@scnu.edu.cn (L. Zhao); jhtao@phy.ecnu.edu.cn (J. Tao)

Photoelectrochemical Measurements

We use the three-electrode method to measure the photocurrent and current/time curve. The working electrode is connected to the photoelectrode and the reference electrode is connected to AgCl. The counter electrode is connected to a platinum plate. Two-electrode method to test the open circuit voltage of TD, the working electrode is connected to the negative electrode of the thermoelectric device, the counter electrode is connected to the positive electrode of the thermoelectric device and the reference counter electrode is empty.

Assuming that the environment is in an ideal state (the container is sealed and there is no heat exchange between the outside and the light is completely absorbed by the aqueous solution. Under one hour (t: 8000 S) of simulated illumination (AM 1.5 G, P: 100 mW / cm^2), the effective light receiving area (S) of the water (specific heat capacity (C): 4.2×10^3 J/Kg K, volume: 125 cm^3, Mass (m): 0.125 Kg) is 25 cm^2, and temperature rises by about 12 K. The rest energy was converted into electrical energy by thermoelectric devices (internal resistance (R), 1.58 Ω).

According to the law of conservation of energy and Joule's law, calculate the ideal thermoelectric voltage, and finally verify the STH efficiency under this ideal thermoelectric voltage through experimental verification.

$$Ideal\ state\ voltage\ (v)\ =\ \sqrt{\frac{(P\times S\times t-C\times m\times \Delta T)\times R}{t}}$$

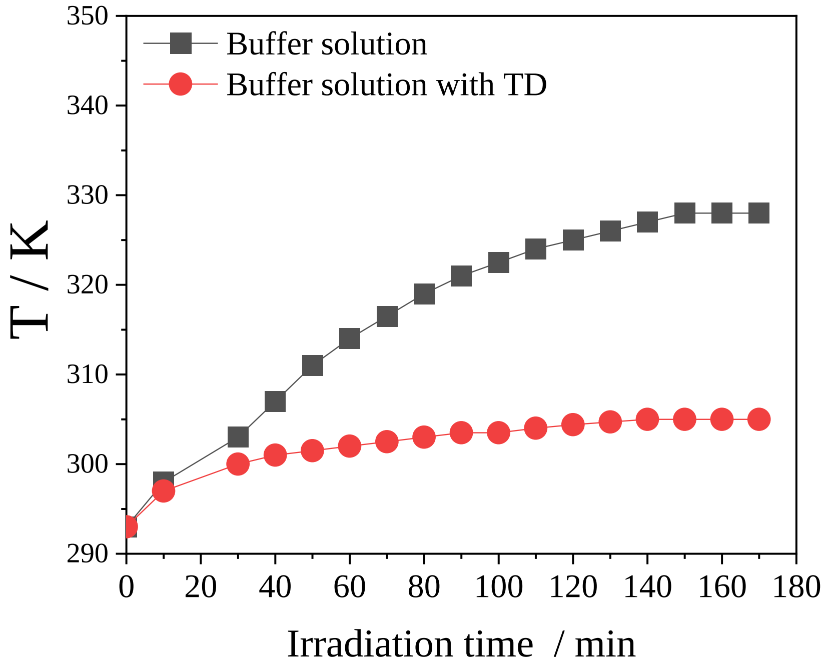

Fig. S1 The temperature curve of the buffer solution with or without TD under solar simulated AM 1.5 G irradiation.

Infrared light accounts for approximately 50% of solar radiation energy. However, the infrared light is normally absorbed in the form of thermal energy rather than photons. As shown in **Fig. S1**, in approximately 160 minutes, the temperature of the buffer solution rose from 293 K to 328 K under solar simulated AM 1.5 G irradiation, and then reached the equilibrium point. This part of the high temperature is caused by the infrared light in the sunlight, but the infrared light cannot be absorbed by the photoelectrode, in our previous study, this part of the heat energy was not fully utilized. In this study, in order to maximize the utilization of the full spectrum of the sunlight, we integrated TD system to convert this part of the thermal energy into electrical energy. We found that with the integration of the TD system, in approximately 130 minutes, the temperature of the buffer solution was finally maintained at about 305 K. The TD system not only produced a significant additional thermoelectric bias of approximately 1.0 V on a Cu_2ZnSnS_4-$BiVO_4$ tandem cell to direct solar water splitting but also cooled the surface temperature of the Cu_2ZnSnS_4 and $BiVO_4$ photoelectrodes to avoid any surface damage caused by solar thermal treatment.

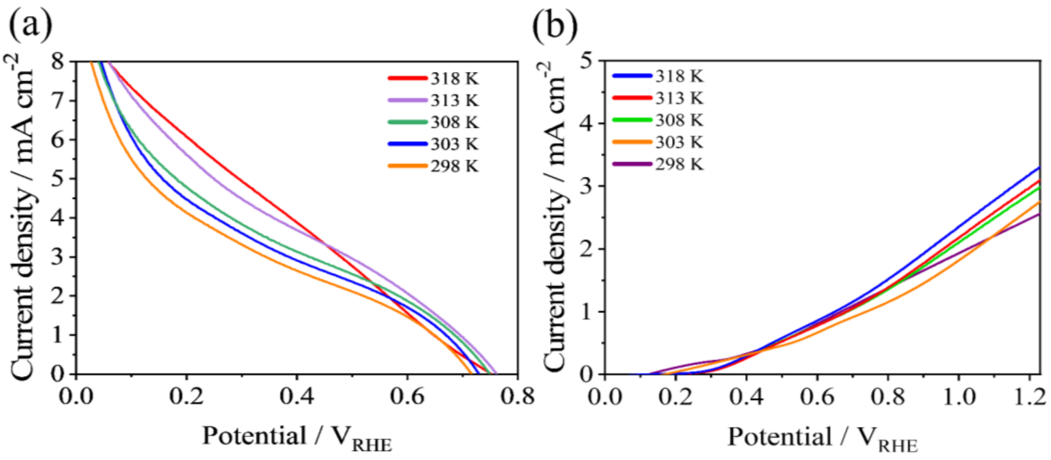

Fig. S2 J-V curves of the CZTS-based photocathode (a) and $BiVO_4$ photoanode (b) at different temperatures. above measurements were carried out in 0.2 mol/L Na_2HPO_4/NaH_2PO_4 solution (pH 6.8) under solar simulated AM 1.5 G irradiation.

In order to test the effect of electrolyte temperature on water splitting, we tested the PEC performance of the photoelectrode in different temperature ranges. **Fig. S2** shows that the current density of CZTS-based photocathode and $BiVO_4$ photoanode increased with increasing temperature. It was found that the PEC performance of CZTS-based photocathode and $BiVO_4$ photoanode slightly increased with temperature. The increment in temperature enhanced the activity of the catalyst and improved the charge transfer speed at the photoelectrode interface.[1] Similar results were already reported.[2-3] However, by comparing with **Fig. 6** and **7**, we can think that the improved PEC performance caused by temperature change is negligible compared with the improvement of photoelectrode performance after integrating thermoelectric device.

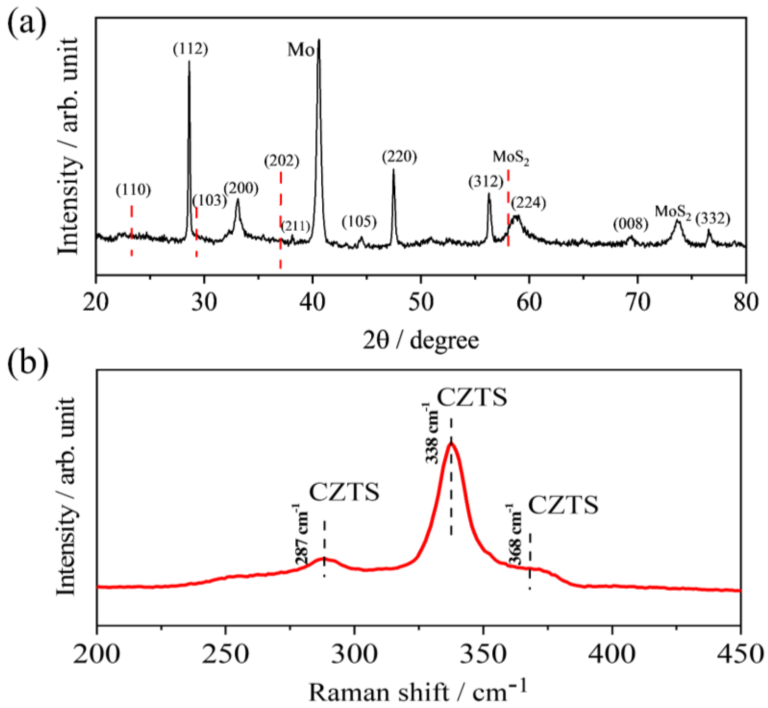

Fig. S3 XRD (a) and Raman spectrum (b) of the CZTS/Mo/glass samples.

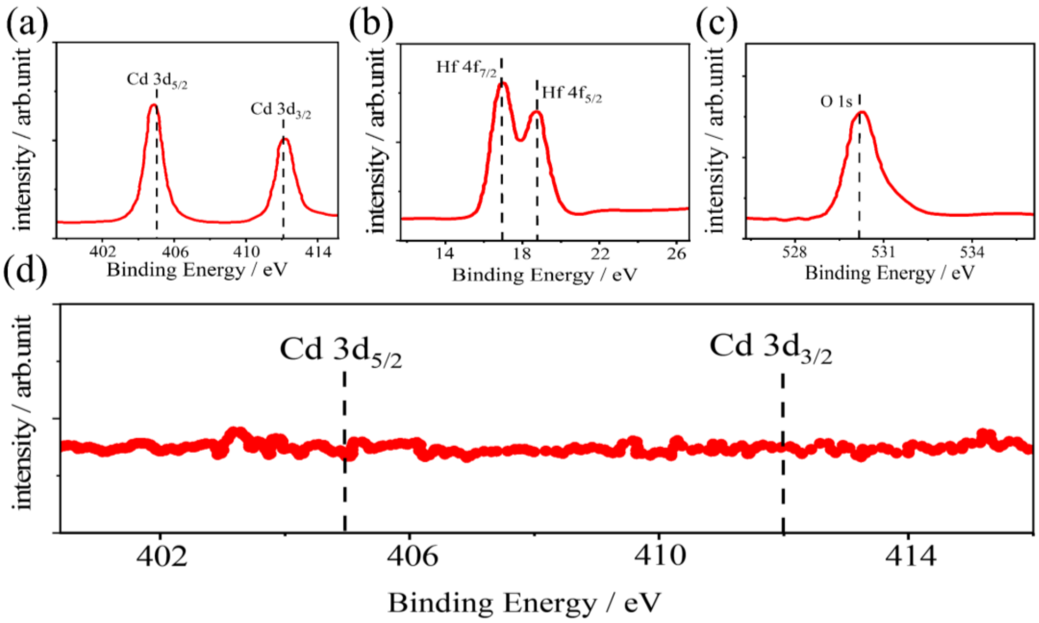

Fig. S4 XPS spectra of Cd 3d, Hf 4f and O 1s of CdS/CZTS (a) and HfO_2/CdS/CZTS (b,c,d) films.

In order to further confirm whether CdS and HfO_2 form an effective modification on the CZTS surface, we tested the XPS spectra of Cd 3d, O 1s and Hf 4f of CdS/CZTS and HfO_2/CdS/CZTS films respectively, and also compared it with the standard values. As shown in **Fig. S4a**, we observed clear Cd $3d_{5/2}$ and Cd $3d_{3/2}$ peaks in CdS/CZTS sample after CdS deposition on the CZTS surface, indicating that CdS has been effectively deposited on the surface of CZTS. When the CdS/CZTS film was covered by loaded HfO_2 layer, we can clearly observe Hf $4f_{7/2}$, Hf $4f_{5/2}$ and O 1s peaks on the surface of HfO_2/ CdS/CZTS (**Fig. S4b** and **c)**, but no Cd peak can be observed (**Fig. S4d)**, indicating the HfO_2 layer entirely covered the surface of CdS/CZTS film.

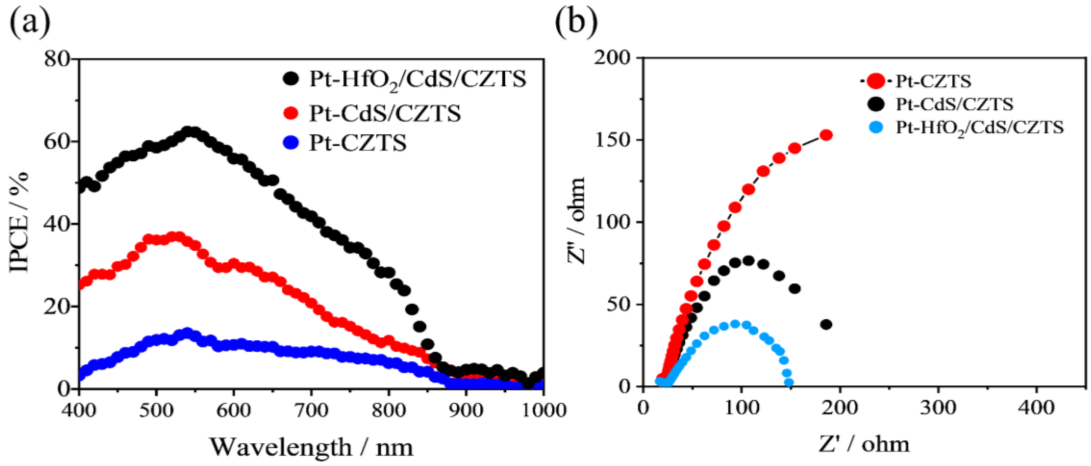

Fig. S5 IPCE curves (a) and EIS curves (b) of Pt-CZTS, Pt-CdS/CZTS, Pt-HfO_2/CdS/CZTS) photocathodes. Above measurements were carried out in 0.2 mol/L Na_2HPO_4/NaH_2PO_4 solution (pH 6.8) under solar simulated AM 1.5 G irradiation.

As shown in **Fig. S5a**, we can clearly see that the deposited CdS and HfO_2 buffer layers can effectively improve the incident photon to current efficiency (IPCE) of CZTS photocathode, and the HfO_2/CdS/CZTS photoelectrode has higher incident photon to current efficiency than bare CZTS. This is because the CdS layer can form a PN junction with the CZTS absorption layer, which can effectively improve the separation efficiency of photogenerated charges in the semiconductor. At the same time, the HfO_2 protective layer deposited on the CZTS can effectively passivate the interface and inhibit the photoelectrode surface photocorrosion, which further reduce the resistance and improve photon absorption efficiency (**Fig. S5b**). The trend in EIS curves also similar with their PEC performances as shown in **Fig. 4**.

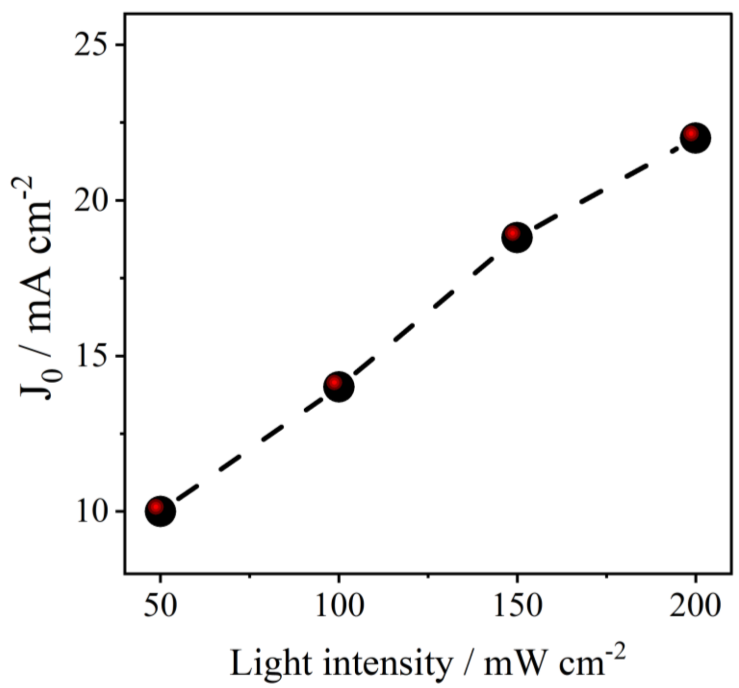

Fig. S6 J_0 (current density at 0 V_{RHE}) curves of Pt/HfO_2/CdS/CZTS photoelectrode at different light intensity. Above measurements were carried out in 0.2 mol/L Na_2HPO_4/NaH_2PO_4 solution (pH 6.8) under solar simulated AM 1.5 G irradiation.

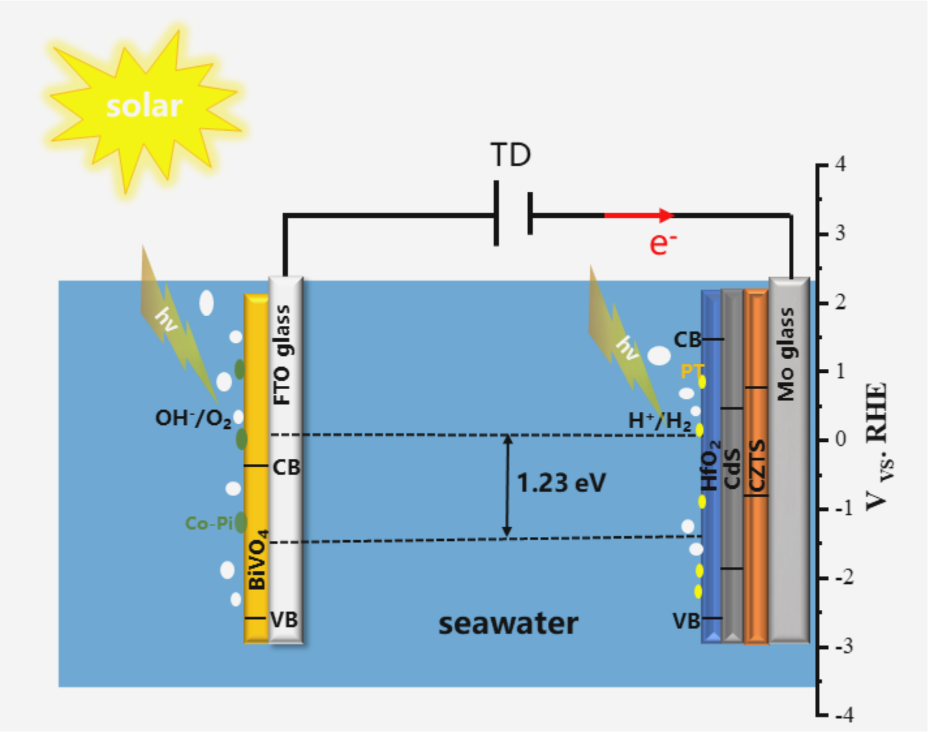

Fig. S7 band diagram of Pt/HfO_2/CdS/CZTS for solar water splitting. The band positions of CZTS, CdS and HfO_2 were reference to previous publications. [4-5]

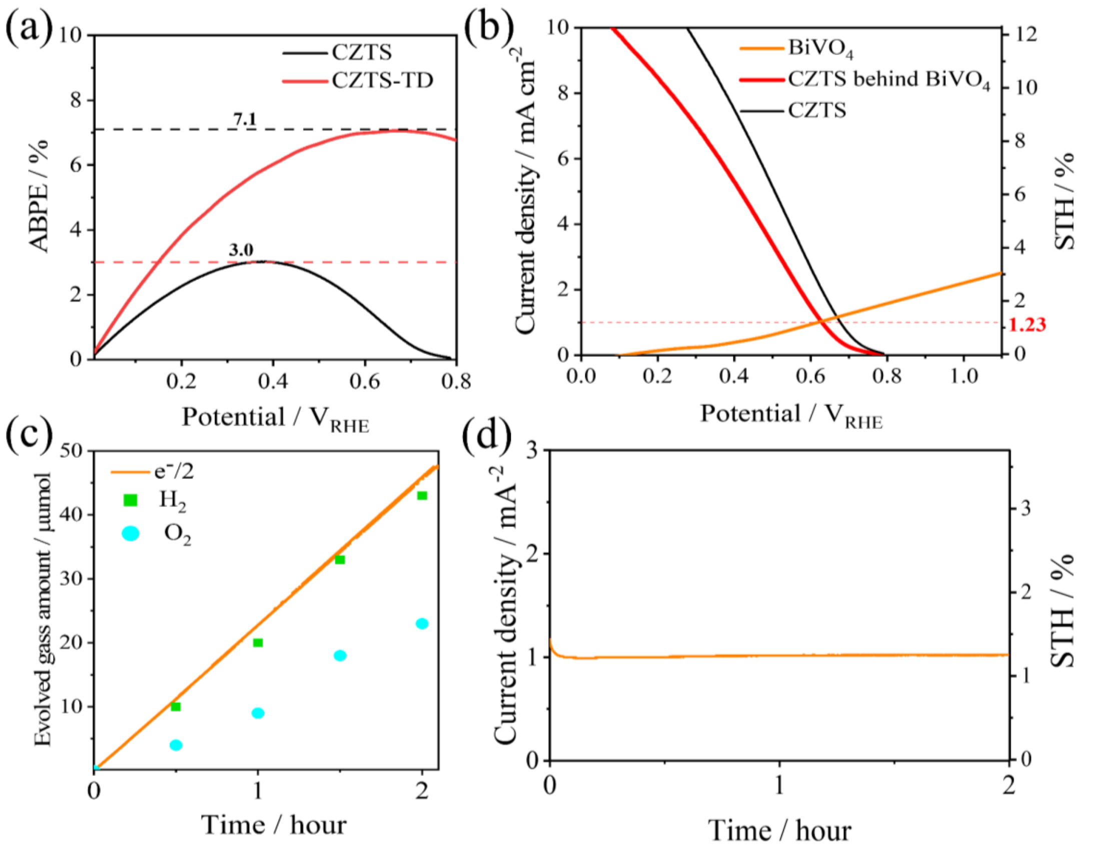

Fig. S8 ABPE curves of CZTS photocathodes with and without TD (a); J-V curves of CZTS photocathode, $BiVO_4$ photoanode and CZTS photocathode behind the $BiVO_4$ photoanode (b); hydrogen and oxygen evolution amount-time curves (hydrogen and oxygen evolution produced from the tandem device detected by gas chromatography, the solid line denotes the time course curve of $e^-/2$) of CZTS-$BiVO_4$ tandem cell (c); photocurrent density-time curve of the CZTS-$BiVO_4$ tandem cell (d). above measurements were carried out in 0.2 mol/L Na_2HPO_4/NaH_2PO_4 solution (pH 6.8) under solar simulated AM 1.5 G irradiation.

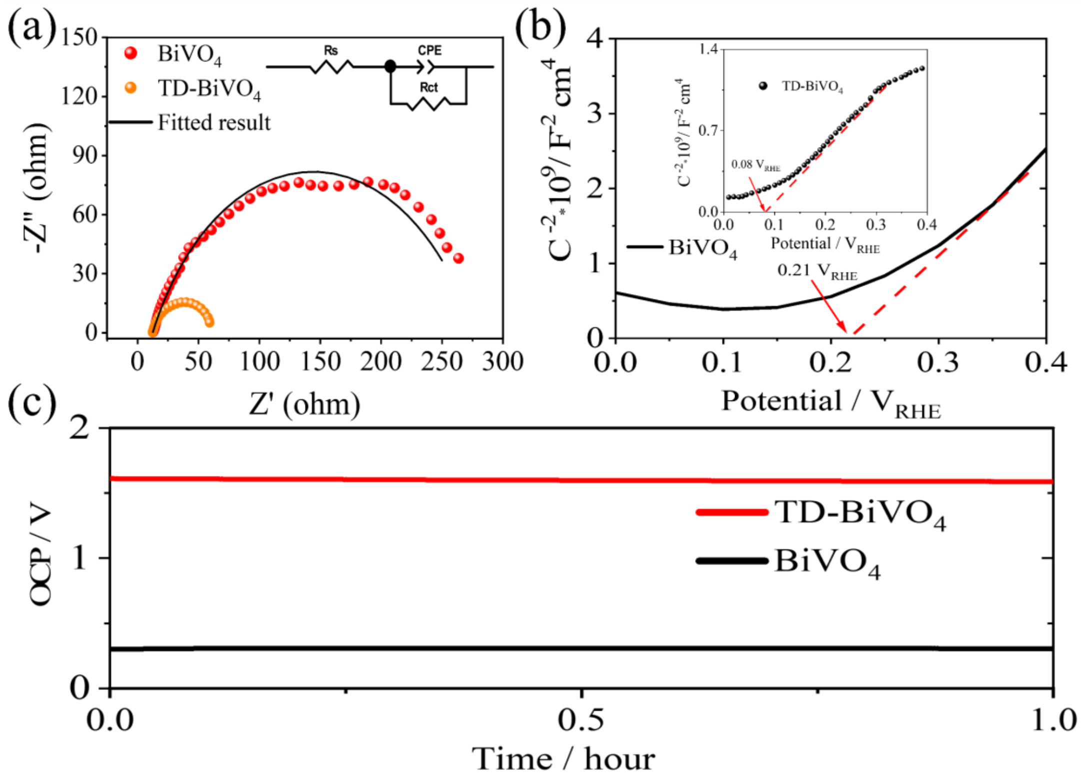

Fig. S9 EIS curves of $BiVO_4$ and TD-$BiVO_4$ (inset: equivalent circuit) (a); M-S plots of $BiVO_4$ and TD-$BiVO_4$ (b); Open circuit potential (OCP) of $BiVO_4$ and TD-$BiVO_4$ (C).

In order to further study the effect of TD on the charge separation process and energy band potential of $BiVO_4$, we tested the EIS, OCP and M-S of $BiVO_4$ and TD-$BiVO_4$, respectively. **Fig. S9a** shows the EIS curves of $BiVO_4$ and TD-$BiVO_4$, the dots in the EIS curve represent the experimental data, and the solid line represents the fitting result according to the equivalent circuit model (inset: equivalent circuit), Rs and Rct in the equivalent circuit diagram represent series resistance and interface charge transfer resistance, respectively.[6] The fitted values of each components are shown in **Table S1**. The Rs of $BiVO_4$ and TD-$BiVO_4$ are similar, indicating that the effect of series resistance is negligible.[7] After integrating the TD, the Rct of TD-$BiVO_4$ is significantly reduced, indicating that the voltage provided by the TD effectively promotes the charge separation and transfer efficiency, thereby enhancing the photocurrent density of $BiVO_4$. As shown in **Fig. S9b**, the M-S plots of $BiVO_4$ and TD-$BiVO_4$ exhibited the positive expected slope, indicating that the applied thermoelectric voltage did not change the hole conductivity type of $BiVO_4$, and the flat-band potential of $BiVO_4$ negatively shifts from 0.21 V to 0.08 V, which is due to

the presence of a large number of surface states on the $BiVO_4$ surface and the reduction of the surface Fermi level pinning effect caused by the applied voltage.[8] Finally, we tested the Open circuit potential (OCP) of $BiVO_4$ and TD-$BiVO_4$. As shown in **Fig. S9c,** with the aid of the TD, the OCP of $BiVO_4$ was increased from 0.3 V to 1.6 V, the high OCP is beneficial to improve the efficiency of water splitting, indicating that the thermoelectric device further optimized the PEC performance of $BiVO_4$.

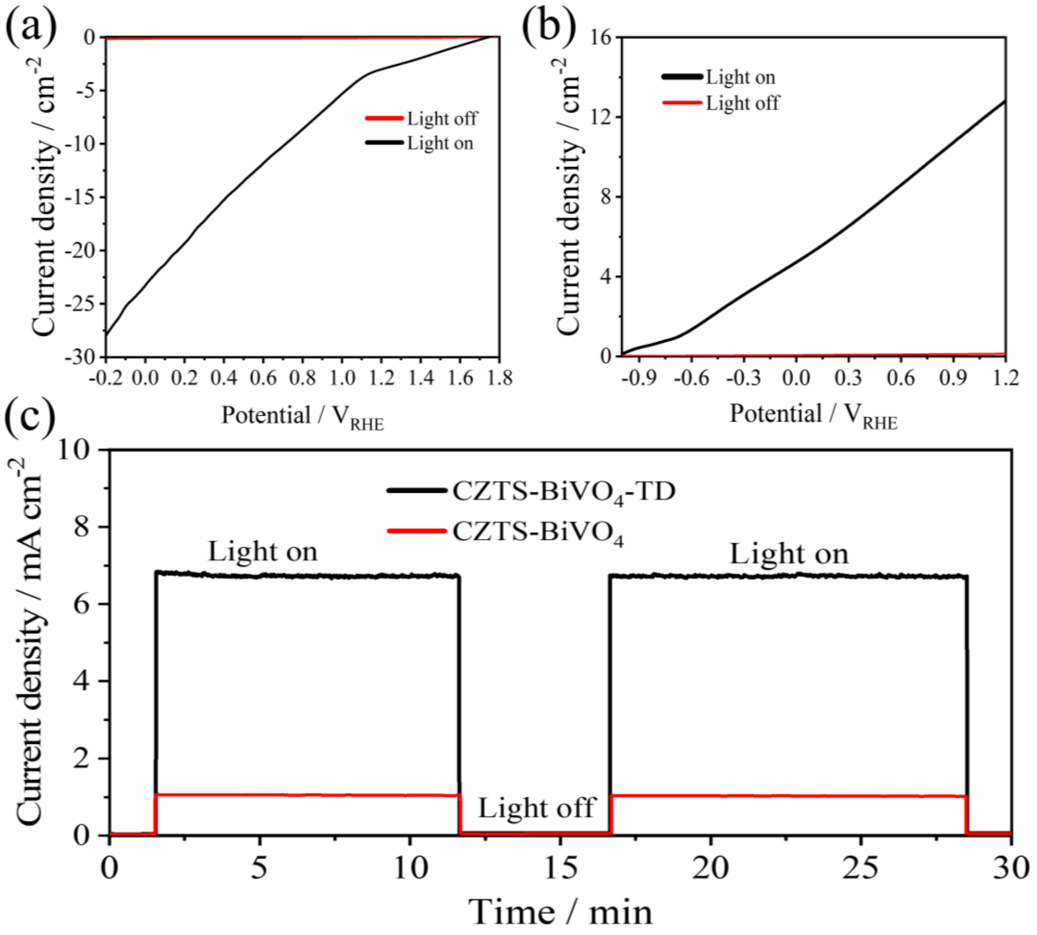

Fig. S10 J-V curves of the TD-CZTS photocathode (a) and TD-$BiVO_4$ photoanode (b)*;* the chopped i-t curves of TD-CZTS-$BiVO_4$ and CZTS-$BiVO_4$ tandem cell (c). Above measurements were carried out in 0.2 mol/L Na_2HPO_4/NaH_2PO_4 solution (pH 6.8) under solar simulated AM 1.5 G irradiation.

In order to test the effect of illumination on the performance of the TD-photoelectrode, we tested the J-V change of the TD-CZTS photocathode and TD-$BiVO_4$ photoanode under the different condition that the simulated light source was turned on/off. As shown in **Fig. S10a**, the photocurrent density of the TD-CZTS photocathode reached about 23 mA/cm^2 (0 V_{RHE}) when the light source is turned on. While the dark current is close to zero when light is turned off. **Fig. S10b** shows the photocurrent density of the TD-$BiVO_4$ photoanode is 13 mA/cm^2 (1.23 V_{RHE}). **Fig. S10c** shown the photocurrent density of the CZTS-$BiVO_4$ tandem cell under the TD-drive can reach about 6.6 mA/cm^2 during the hydrogen production process, which is a very significant improvement compared to the cell without TD-drive, which is only

about 1 mA/cm^2. It is worth noting that the photocurrent of the CZTS-$BiVO_4$ tandem cell is 0 mA/cm^2 regardless of whether there is TD or not. This is because the voltage provided by the TD (about 1 eV) is much smaller than the band gap of CZTS (1.45 eV) and $BiVO_4$ (2.3~2.4 eV). In the absence of sunlight, the electrons in the semiconductor cannot obtain enough energy through photoexcitation and transition to the conduction band, so conduction cannot be formed, and the entire TD-CZTS-$BiVO_4$ tandem cell circuit is in a disconnected state.

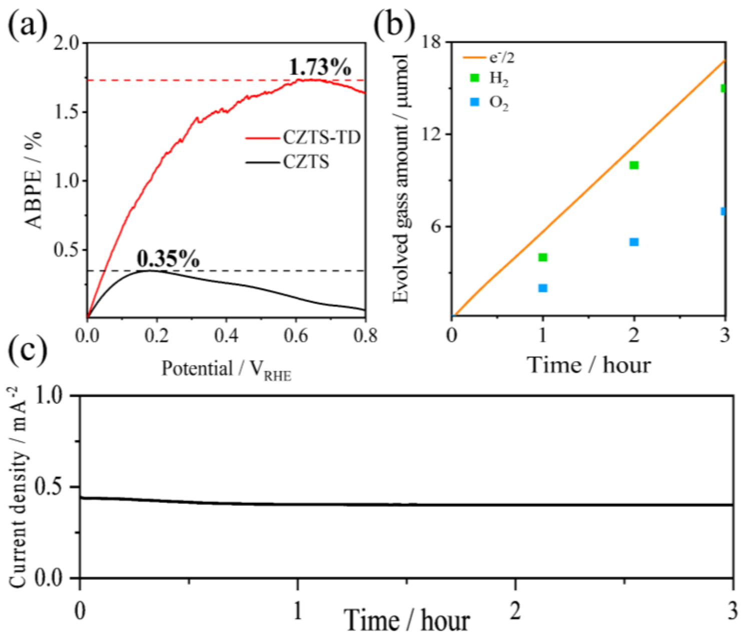

Fig. S11: ABPE curves of CZTS-based photocathode with and without TD (a); hydrogen and oxygen evolution amount-time curves (hydrogen and oxygen evolution produced from the tandem device detected by gas chromatography, the solid line denotes the time course curve of $e^-/2$) of the CZTS-$BiVO_4$ tandem cell (b); photocurrent density-time curve of the CZTS-$BiVO_4$ tandem cell (c). Above measurements were carried out in natural seawater (pH 7.9) under solar simulated AM 1.5 G irradiation.

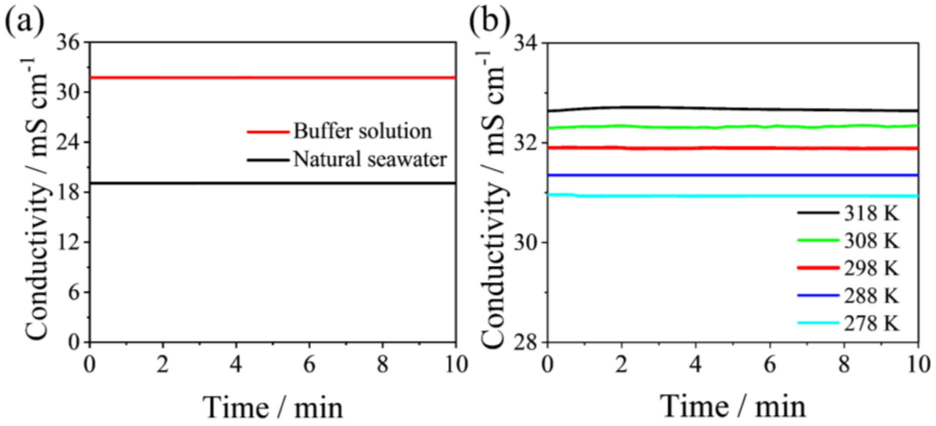

Fig. S12: Comparison of conductivity between buffer solution (0.2 mol/L Na_2HPO_4/NaH_2PO_4 solution; pH 6.8) and natural seawater (a); Conductivity test under different temperature gradients (b).

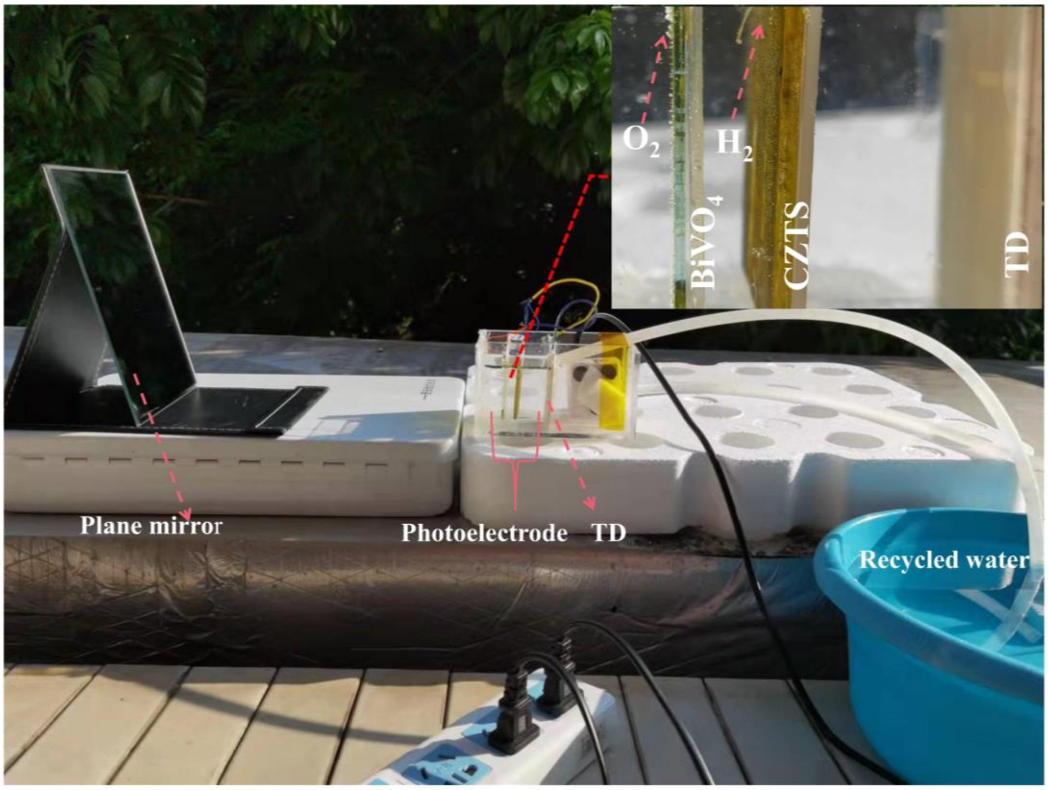

Fig. S13: photograph of the TD-CZTS-BiVO$_4$ tandem devices under outdoor sunlight irradiation.

Table S1

Impedance fit values for each component of $BiVO_4$ and TD-$BiVO_4$.

$BiVO_4$			
**Element**	Value	Error	Error %
**Rs**	12.46	0.079642	0.63918
**CPE-T**	0.00019306	5.0701E-6	2.6262
**CPE-P**	0.70777	0.0039072	0.55204
**Rct**	263.2	2.8359	1.0775

TD-$BiVO_4$			
**Element**	Value	Error	Error %
**Rs**	12.1	0.049346	0.40782
**CPE-T**	0.0012028	3.8468E-5	3.1982
**CPE-P**	0.68072	0.0056263	0.82652
**Rct**	51.32	0.52652	1.026

Table S2.

Chemical reaction formula and Normal Hydrogen Electrode (NHE) under different conditions.

Hydrogen Evolution Reaction (298 k)		Oxygen Evolution Reaction (298 k)	
Acidic conditions<br>0.2 mol/L Na_2HPO_4/NaH_2PO_4 solution (pH 6.8)		Acidic conditions<br>0.2 mol/L Na_2HPO_4/NaH_2PO_4 solution (pH 6.8)	
**$2H^+ + 2e^- \rightarrow H_2$**	**NHE**= 0.00 V	**$2H_2O \rightarrow O_2+4H^++4e^-$**	**NHE**= -1.23 V
Alkaline conditions (seawater, pH 8.0)		Alkaline conditions (seawater, pH 8.0)	
**$2H_2O+2e^- \rightarrow H_2+2OH^-$**	**NHE**= -0.83 V	**$4OH^- \rightarrow O_2+2H_2O+4e^-$**	**NHE**= -0.40 V

Table S3

Comparison of main ions in natural seawater and buffer solution (ion concentration exceeds 100 mg/L).

ion concentration (mg/L)	Na^+	Cl^-	H^+	Mg^{2+}	HPO_4^{2-}
natural seawater	1350	2350	#	184	#
buffer solution	13800	#	200	#	38400
#; Not counted in statistics					

References

[1] B. He, S. Jia, M. Zhao, Y. Wang, T. Chen, S. Zhao, Z. Li, Z. Lin, Y. Zhao, X. Liu, *Adv Mater.* **2021**, *33*, e2004406.

[2] X. Ye, J. Yang, M. Boloor, N. A. Melosh, W. C. Chueh, *J. Mater. Chem. A.* **2015**, *3*, 10801.

[3] L. Zhang, L. Sun, Z. Guan, S. Lee, Y. Li, H. D. Deng, Y. Li, N. L. Ahlborg, M. Boloor, N. A. Melosh, W. C. Chueh, *Nano Lett.* **2017**, *17*, 5264.

[4] M. P. Suryawanshi, S. W. Shin, U. V. Ghorpade, K. V. Gurav, C. W. Hong, G. L. Agawane, S. A. Vanalakar, J. H. Moon, J. H. Yun, P. S. Patil, J. H. Kim, A. V. Moholkar, *Electrochim. Acta.* **2014**, *150*, 136.

[5] Z. Xing, F. Ren, H. Wu, L. Wu, X. Wang, J. Wang, D. Wan, G. Zhang, C. Jiang, *Sci. Rep.* **2017**, *7*, 43901.

[6] X. Lv, K. Nie, H. Lan, X. Li, Y. Li, X. Sun, J. Zhong, S.-T. Lee, *Nano Energy.* **2017**, *32*, 526.

[7] B. Klahr, S. Gimenez, F. Fabregat-Santiago, J. Bisquert, T. W. Hamann, *J Am Chem Soc.* **2012**, *134*, 16693.

[8] C. Du, X. Yang, M. Mayer, H. Hoyt, J. Xie, G. McMahon, G. Bischoping, D. Wang, *Angewandte Chemie (International ed. in English).* **2013**, *52*, 12692.
